# Thioester‐Based Coupled Fluorogenic Assays in Microdevice for the Detection of Single‐Molecule Enzyme Activities of Esterases with Specified Substrate Recognition

**DOI:** 10.1002/advs.202306559

**Published:** 2023-12-22

**Authors:** Tatsuya Ukegawa, Toru Komatsu, Mayano Minoda, Takuya Matsumoto, Takumi Iwasaka, Tadahaya Mizuno, Ryo Tachibana, Shingo Sakamoto, Kenjiro Hanaoka, Hiroyuki Kusuhara, Kazufumi Honda, Rikiya Watanabe, Yasuteru Urano

**Affiliations:** ^1^ Graduate School of Pharmaceutical Sciences The University of Tokyo 7‐3‐1 Hongo, Bunkyo‐ku Tokyo 113‐0033 Japan; ^2^ Graduate School of Pharmaceutical Sciences Keio University 1‐5‐30, Shibakoen, Minato‐ku Tokyo 105–8512 Japan; ^3^ Graduate School of Medicine Nippon Medical School 1‐1‐5 Sendagi, Bunkyo‐ku Tokyo 113–8602 Japan; ^4^ Institute for Advanced Medical Science Nippon Medical School 1‐1‐5 Sendagi, Bunkyo‐ku Tokyo 113–8602 Japan; ^5^ Cluster for Pioneering Research Riken, 2‐1 Hirosawa, Wako Saitama 351‐0198 Japan; ^6^ Graduate School of Medicine The University of Tokyo 7‐3‐1 Hongo, Bunkyo‐ku Tokyo 113‐0033 Japan

**Keywords:** chemical biology, enzymes, enzymomics, single‐molecule analysis

## Abstract

Single‐molecule enzyme activity assay is a platform that enables the analysis of enzyme activities at single proteoform level. The limitation of the targetable enzymes is the major drawback of the assay, but the general assay platform is reported to study single‐molecule enzyme activities of esterases based on the coupled assay using thioesters as substrate analogues. The coupled assay is realized by developing highly water‐soluble thiol‐reacting probes based on phosphonate‐substituted boron dipyrromethene (BODIPY). The system enables the detection of cholinesterase activities in blood samples at single‐molecule level, and it is shown that the dissecting alterations of single‐molecule esterase activities can serve as an informative platform for activity‐based diagnosis.

## Graphical abstract

The single‐molecule enzyme activity assay platform is reported for esterases with strict substrate recognition utilizing the coupled assay using thioesters as substrate analogues. The highly water‐soluble thiol‐reacting probes are developed based on phosphonate‐substituted boron dipyrromethene (BODIPY), which enables the detection of cholinesterase activities in blood samples at single‐molecule level for activity‐based diagnosis.

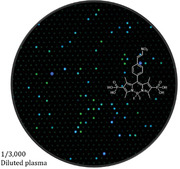

## 1 Introduction

**Figure 1:**
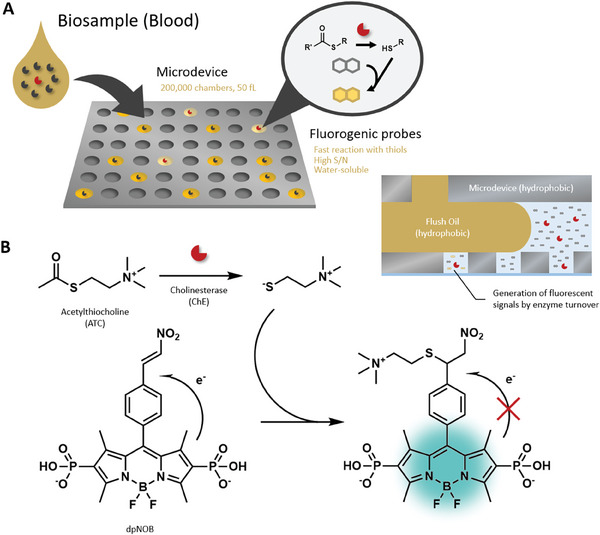
Design of coupled assay to detect thiol‐generating enzyme reactions in microdevice. A) Concept of microfabricated chamber‐based single‐molecule enzyme activity assay. B) Design of coupled assay of thiols using the fluorogenic probe pNOB.

In living systems, there are thousands of enzymes present to maintain the homeostasis, and their functional alterations are often tightly connected the onset of diseases.^[^ ^1^, ^2^, ^3^, ^4^ ^]^ Activities of enzymes are modified under the control of various factors at transcriptional, translational, and posttranslational levels, and directly monitoring the activities of enzymes in living samples can provide the meaningful information that are directly connected to phenotypic changes. Especially, recent progress of the methodologies to study enzymatic activities at single‐molecule level offers a rich source of information for understanding protein functions with proteoform resolution, i.e., at the level of unique protein structural variations occurring from various posttranslational modifications and protein‐protein interactions.^[^ ^3^, ^4^ ^]^ Single‐molecule enzyme activity analysis is performed by preparing for the fluorogenic substrate analogues whose metabolism generates the fluorescent product that accumulates in the microfabricated chamber containing the enzyme (**Figure** **1**A).^[^ ^5^, ^6^ ^]^ In the condition where theoretically 0 or 1 target enzyme is included in each chamber, we can detect the activities of each enzyme molecule independently and simultaneously. The key features of the assay are (1) since the volume of the chamber is small (50 fL in this study), the fluorescence signal can be sufficiently amplified to the detectable level (µm order) even from the turnover of the single‐molecule enzyme (Table S1, Supporting Information), (2) by analyzing large number of chambers (≈160 000 in this study), it can detect the low concentration (fM order) of target proteins (Table S2, Supporting Information), and (3) the assay can detect the activities of individual molecules coming from different proteoforms.^[^ ^6^, ^7^ ^]^ Despite the potential of the assay to be used as a versatile methodology to study enzyme activity profiles at proteoform levels, the limitation of current single‐molecule enzyme activity analysis is the limited availability of the fluorogenic substrates used in the study. The assays have been developed for glycosidases,^[^ ^5^ ^]^ phosphatases^[^ ^6^ ^]^ and peptidases,^[^ ^8^ ^]^ but the expansion of the targetable enzymes is highly desirable.^[^ ^1^ ^]^ One of the obstacles in expanding the target is that the substrate modification with fluorophores abolishes the substrate recognition of enzymes due to the structural perturbation by large size fluorophores. For example, cholinesterases (ChEs), enzymes that metabolize choline esters, is known to recognize the structure of choline, so the proper fluorophore‐modified analogue with sufficient reactivity and selectivity was not easily designed for the enzymes.^[^ ^9^, ^10^ ^]^ In this study, in order to broaden the scopes of the assay, we utilize the thiol‐based coupled assay that uses thioesters as a structural analogue of ester. Sulfur belongs to the same elemental family as oxygen, and the natural substrate analogue whose oxygen atom of ester is converted to sulfur can be tolerated by diverse esterases. The hydrolysis of thioester generates thiol that shows unique reactivities toward certain electrophiles (e.g., Michael acceptors) so they are selectively detectable over alcohol or H_2_O. We considered that, by designing the coupled assay that specifically detect thiol generation in the microfabricated chamber device, we may be able to detect the single‐molecule enzyme activities of various esterases.

## 2 Results and Discussion

### 2.1 Development of Fluorogenic Substrates for Thiol‐Based Coupled Assays to Detect Single‐Molecule Esterase Activities

Currently, the major thiol‐based coupled assay is one using 5,5′‐dithiobis‐(2‐nitrobenzoic acid) (DTNB), but its colorimetric output is not suitable for the assay using microdevice. There are reports on the development of thiol‐reacting fluorogenic probes,^[^ ^11^ ^]^ but in order for the probes to be used in the microdevice‐based single‐molecule enzyme activity analysis, it needs to meet the criteria, (1) good reactivity and selectivity toward thiols, (2) high signal‐to‐noise (S/N) ratio, and (3) sufficient hydrophilicity. (1) and (2) are required since the probe needs to detect the benign activity generated from the turnover of single‐molecule enzyme, and (3) is the key factor for the probe to be used in microdevice, since microdevice and the oil used to seal the device are hydrophobic materials and the hydrophobic compounds suffer from the leakage out of the device (Figure S1, Supporting Information).^[^ ^6^ ^]^ We have designed the phosphonate‐substituted boron dipyrromethene (BODIPY) baring the nitroolefin group as a reaction site serves best for the purpose (Figure 1B).

**Figure 2:**
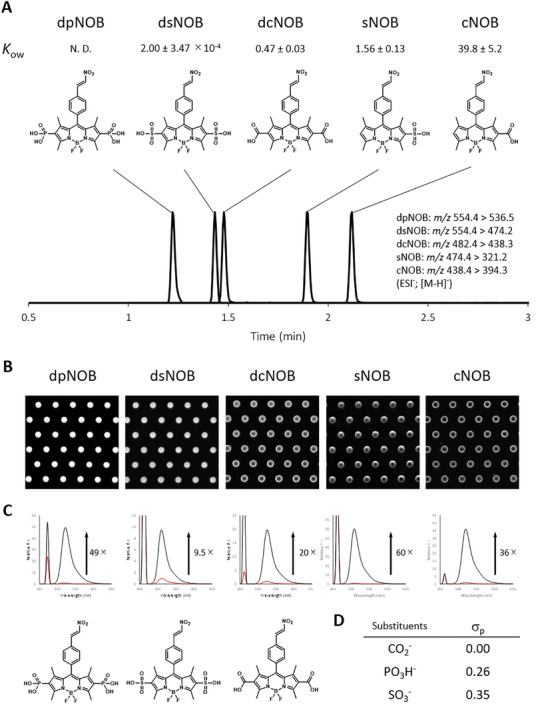
Development of dpNOB as an optimized probe for the detection of thiols in microdevice. A) LC‐MS/MS chromatograms of various BODIPY derivatives separated by reverse phase mode. KOW values of n‐octanol‐water separation are shown for each compound (value ± S. D., *n* = 3). B) Confocal fluorescence images of microdevice loaded with BODIPY derivatives (30 µM) reacted with N‐acetylcysteine (100 µm) in HEPES buffer (100 mm, pH 7.4) containing Triton X‐100 (3 mm). C) Fluorescence spectra of BODIPY derivatives (1 µm) before and after reaction with GSH (100 µm). Number along the arrow indicate the fluorescence activation ratio (after/before). D) Hammett constant σ_p_ of substituents.

As for factor (1) and (2), among many thiol‐reaction sites reported,^[^ ^11^ ^]^ we selected nitroolefin as the best candidate. Currently, many thiol‐reacting fluorescent probes are developed based on *N*‐phenylmaleimide (NPM) as a reaction site.^[^ ^12^ ^]^ The reaction rate of NPM toward thiols (*k* = 1.2 × 10^3^ sec^−1^) was faster than that of nitroolefin (*k* = 78 sec^−1^) (Figure S2, Supporting Information), but NPM‐based probe suffered from the background fluorescence increase from the hydrolysis of maleimide to maleamidic acid (Figures S2 and S3, Supporting Information). In our experimental conditions, nitroolefin was more stable over the same time period (Figure S2, Supporting Information), and we considered that it serves as a reliable reaction site for the probe design. We have developed the probe using BODIPY as a fluorophore that exhibits strong absorbance/fluorescence at visible light region.^[^ ^13^, ^14^ ^]^ The fluorophore has smaller Stokes shift compared to other fluorophores at the same wavelength ranges, such as fluorescein and rhodamine, but we considered that the ease of various chemical modifications for controlling photophysical and chemical characters is advantageous for the functional probe design. We have previously reported that BODIPY directly attached to nitroolefin exhibits the fluorescence quenching by donor‐induced photoinduced electron transfer (d‐PeT).^[^ ^15^, ^16^ ^]^ In this scheme, the electron‐accepting nitroolefin receives single electron from the excited fluorophore, which compete with the fluorescence emission process, so the reaction on nitroolefin to reduce its electron‐accepting capacity can be monitored by fluorescence activation (Figure S4A, Supporting Information). We have originally reported that this probe can monitor the Henry reaction with enol as a nucleophile.^[^ ^15^ ^]^ In this study, we have confirmed that the reaction of thiols with nitroolefin can also be monitored by the probe with more than 50‐times fluorescence activation (Figure S4B, Supporting Information). However, the original nitroolefin‐BODIPY was hardly soluble in aqueous solution, which led to the design of novel probes to fulfill factor (3), i.e., to add an increased hydrophilicity. The introduction of polar functional groups such as carboxylic acid (CO_2_H)^[^ ^17^ ^]^ and sulfonic acid (SO_3_H)^[^ ^18^ ^]^ at 2,6‐position of BODIPY improves the hydrophilicity of the probes. However, when we prepared the nitroolefin‐based probes (**Figure** **2**A; Scheme S1, Supporting Information) and used them in microdevice‐based fluorometric assay, they suffered from several problems. As for 2,6‐CO_2_H‐substituted BODIPY, the hydrophilicity was not sufficient in microdevice‐based assay; when the fluorescent thiol‐adducts of probes were loaded into the microdevice, probes with lower hydrophilicities (i.e., mono‐CO_2_H, di‐CO_2_H, and mono‐SO_3_H‐substituted BODIPY) tended to show the ring‐like fluorescence images coming from the non‐specific binding of the probe to the device (Figure 2B);^[^ ^8^ ^]^ this was problematical since the irregular free dye concentration in the chamber can interfere with the precise quantification of the signal and the binding to the hydrophobic surface can lower the efficiency of PeT to generate the background fluorescence (Figure S1, Supporting Information).^[^ ^19^ ^]^ The probe with two sulfonic acids exhibited sufficient hydrophilicity, but the decrease of S/N of fluorescence signal was observed (Figure 2C). The intact probe showing stronger fluorescence was the major cause of lower S/N, which we considered came from the introduction of highly electron withdrawing sulfonate (SO_3_^−^; σ_p_ = 0.35)^[^ ^20^ ^]^ lowering HOMO energy level and reducing the d‐PeT efficiency toward nitroolefin. Therefore, we searched for another polar substituents and focused on phosphonic acid (PO_3_H_2_) group. Phosphonic acid is less electron withdrawing than sulfonate (PO_3_H^−^; σ_p_ = 0.26) and is expected to dramatically increase the hydrophilicity of the compounds^[^ ^21^ ^]^ (Figure 2D). While various substituents had been introduced into 2,6‐positions of BODIPY^[^ ^16^, ^17^, ^22^, ^23^ ^]^ (Figure S5, Supporting Information), phosphonate‐substituted BODIPY was not reported, and we established the synthetic route of the 2,6‐diphosphono‐BODIPY based on the modified Knorr pyrrole synthesis^[^ ^24^ ^]^ (Scheme S2, Supporting Information). Diethyl (2‐oxopropyl)phosphonate was reacted with ethyl 2‐oximinoacetoacetate to yield the pyrrole scaffold with diethyl phosphonate at desired position. Then, *tert*‐butyl ester of carboxylic acid was cleaved by trifluoroacetic acid and subsequent decarboxylation under acidic condition generated the desired pyrrole. The pyrrole was subject to oxidation, so the pyrrole generated in situ under Ar atmosphere was readily reacted with benzaldehyde to generate desired dipyrromethene.

Firstly, we synthesized the 2,6‐diphosphono‐BODIPY without nitroolefin substitution (dpBODIPY) to study its photochemical characters. dpBODIPY showed absorbance peaked at 498 and 510 nm and fluorescence peaked at 512 and 525 nm in aqueous media with varied pH (Figure S6, Supporting Information). It seemed that the presence of two absorbance/fluorescence peaks came from the equilibrium of phosphonate group (PO_3_^2−^ and PO_3_H^−^); apparent p*K*_a_ was calculated to be 7.3. In acidic condition (pH 5, major molecular form is considered to be PO_3_H^−^), quantum yield was 0.79, and in basic condition (pH 9, major molecular form is considered to be PO_3_^2−^), quantum yield was 0.57, so both PO_3_^2−^ and PO_3_H^−^ forms were strongly fluorescent. Then, the scaffold was used to develop the thiol‐reacting probe utilizing the nitroolefin. The probe dpNOB was highly hydrophilic compared to other BODIPY derivatives. Retention in reverse phase column chromatography is used as the estimation of the hydrophilicity,^[^ ^25^ ^]^ and dpNOB showed the shortest retention time over disulfonated or dicarboxylated derivatives (Figure 2A), indicating that dpNOB was more hydrophilic than the present BODIPY derivatives. The same trend was confirmed in *n*‐octanol‐water separation (Figure 2A). dpNOB showed a strong fluorescence activation upon reaction with thiols (Figure 2C; Figure S7, Supporting Information) and the reaction product showed the good retention in the device (Figure 2B). The validity of the design was supported by the computational calculations (Table S3, Supporting Information). HOMO energy levels of the fluorophores were calculated to estimate the subjectivity toward d‐PeT, and 2,6‐disulfonate BODIPY that showed the increased background fluorescence had the lowest HOMO energy level, while estimated HOMO energy level of 2,6‐diphosphonate BODIPY in its dianionic form was higher than that, indicating the d‐PeT process can occur more efficiently.^[^ ^16^ ^]^ Solvation free energies were calculated with nitroolefin derivatives to estimate the hydrophilicity of compounds and affinity toward hydrophobic solvents, and highest negative solvation free energy of dpNOB (vs air or Et_2_O) supported the strong hydrophilicity of the probes. Overall, dpNOB was considered to be ideal to design coupled assay with good reactivity with thiols, sufficient S/N, and high hydrophilicity.

### 2.2 Single‐Molecule Enzyme Activity Assay of Cholinesterases using pNOB

**Figure 3:**
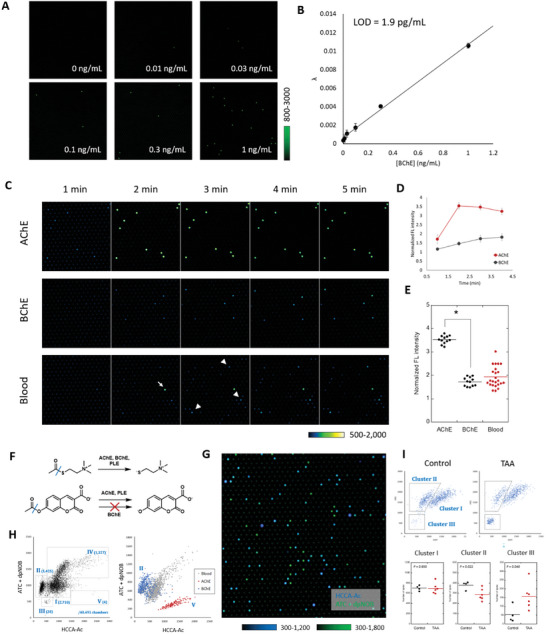
Detection of single‐molecule activities of cholinesterases. A) Epifluorescence images of microdevice containing recombinant BChE (from equine serum, 0–1 ng mL^−1^) with dpNOB (30 µm) and achetylthiocholine iodide (ATC, 1 mm) in HEPES Buffer (10 mm, pH 7.4, containing 0.1% CHAPS). Images were acquired after 30 min incubation at 25˚C. B) l (existence probability per a well) values observed in the detection of varied concentrations of BChE in the same condition as (A). Error bars represent S. D. (*n* = 3). Limit of detection (LOD) was calculated as the value corresponding to 3.29 σ. C) Epifluorescence images of microdevice containing AChE or BChE (1 ng mL^−1^), or human plasma (1/3000) with fluorescence probes (30 µm) with or without ATC (1 mm) in HEPES Buffer (10 mm, pH 7.4, containing 0.1% CHAPS), after incubation for 1–5 min at 25˚C. D) Quantification of fluorescence intensities of spots containing AChE or BChE. Error bars represent S. D. (*n* = 12). The signal was normalized to the signal of empty wells. E) Normalized fluorescence intensities of spots containing enzymes in recombinant AChE or BChE or 1/3000 diluted human plasma samples. *n* = 12. *P < 0.05 (Student's *t*‐test). F) Metabolism of ATC and HCCA‐Ac by various esterases. G) Overlayed confocal fluorescence images of microdevice loaded with 1/3000 diluted human plasma samples mixed with ATC (1 mm), dpNOB (30 µm), and HCCA‐Ac (100 µm) and incubated for 2 h. Blue fluorescence signal corresponds to that of HCCA‐Ac (DAPI filter) and green fluorescence signal corresponds to that of ATC + dpNOB (FITC filter). Individual images are shown in Figure S9A (Supporting Information). H) (Right) Scattered plot of fluorescence intensities of spots with the activities. The horizontal axis refers to the signal at DAPI channel, and the vertical axis refers to the signal at FITC channel. The clusters were marked as I‐V, and the number of spots observed in the cluster was shown in blue letters. The plot was constructed from the images containing 40491 chambers and the incubation time was 2 h. (Left) Overlay of scattered plots generated from the analysis of recombinant AChE (red), recombinant BChE (blue) and 1/3000 diluted plasma samples. The plot was constructed from the images containing ≈10 000 chambers and the incubation time was 1.5 h. I) (Top) Representative scattered plots generated from the analysis of 1/3000 diluted plasma of mice treated with or without TAA for acute liver damage model. Histograms of individual mice were shown in Figure S11 (Supporting Information). For the assay, 1/3000 diluted blood was mixed with ATC (1 mm), dpNOB (30 µm), and HCCA‐Ac (100 µm) and incubated at 25˚C for 2 h. (Bottom) Analysis of the number of molecules in cluster I‐III in blood samples of control or TAA‐treated mice. *n* = 4 for control mice and *n* = 6 for TAA‐treated mice. *P* values were calculated using Student's *t*‐test.

Then, we have used dpNOB to study the single‐molecule enzyme activities of ChEs. We have confirmed that both acetylcholinesterase (AChE) and butyrylcholinesterase (BChE) reacted with acetylthiocholine (ATC), sulfur‐substituted version of acetylcholine, along with other esterases such as porcine liver esterase (PLE) (Figure S8, Supporting Information). Then, we have utilized this coupled assay to detect the single‐molecule activities of enzymes in the microdevice. After the enzyme solution was mixed with ATC and dpNOB and loaded into the device, the fluorescent spots were detected, and the number of spots reflected the concentration of the enzyme molecules (**Figure** **3**A,B), which showed that the probe was able to detect the single‐molecule ChE activities in the microdevice. The limit of detection (LOD) of the enzyme was calculated to be 1.6 pg mL^−1^. The value was consistent with the theoretical expectation (Table S1, Supporting Information) and was higher than those observed in conventional assays (LOD = 350 pg mL^−1^, Figure S9, Supporting Information).

An advantage of single‐molecule enzyme activity assay is that we can independently detect the different proteoforms in the mixture of enzymes. AChE has higher turnover number toward ATC than BChE (*k*_cat_ = 6500 for AChE^[^ ^26^ ^]^ and 336 for BChE^[^ ^27^ ^]^), and we considered that AChE and BChE could be separately detectable in the single‐molecule activity assay based on the different kinetics (Table S1, Supporting Information); the fluorescence signal of chambers containing AChE reached to plateau within 2 min, while the signals from BChE kept increasing over 5 min. When we monitored the fluorescence at 2 min, the fluorescent signals of AChE and BChE were separately detected (Figure 3C–E; Figure S10, Supporting Information). In application of coupled assays toward biological samples, the signal can be affected by the metabolites included in the sample.^[^ ^28^ ^]^ In this case, thiol‐containing metabolites such as cysteine and glutathione (GSH) can be problematical, and we have evaluated the effect by adding varied concentrations of GSH to recombinant BChE and detecting the activities (Figure S11, Supporting Information). The single‐molecule enzyme assays was less subject to the interference, since (1) the background fluorescence increase occurs evenly to all chambers, so the calculation of the enzyme concentrations by counting the activity spots was not dramatically affected, and (2) the assay can be performed with the diluted conditions compared with the conventional assays due to the higher detection sensitivity.

### 2.3 Analysis of Single‐Molecule Esterase Activities in Blood Samples as a Platform of Activity‐Based Diagnosis

After successful detection of the activities of recombinant enzymes, we have extended the study to test if the enzyme activities were detectable in blood samples that contain numerous numbers of proteins. In 1/3000‐diluted blood samples from healthy subjects, the activity spots were detected in the presence of ATC (Figure 3C; Figure S12, Supporting Information). The single molecule activities showed wider distribution than that can be explained by the simple combination of AChE and BChE activities (Figure 3E). The result implied that diverse esterases, not only AChE and BChE, in blood samples contributes to the metabolism of ATC, and we tried to dissect them using the multi‐colored single‐molecule enzyme activity assays. In this assay scheme, the multi‐colored substrates targeting related enzymes were prepared, and the different enzyme species were separately detected by the different reactivity preferences toward them.^[^ ^6^ ^]^ For the purpose, 7‐hydroxycoumarin‐3‐carboxylic acid (HCCA), a fluorophore that has excitation and emission at shorter wavelengths than BODIPY, was acetylated to prepare the fluorogenic substrate analogue toward general esterases^[^ ^29^ ^]^ (Figure 3F). In in vitro assays, HCCA‐Ac reacted with AChE and PLE but did not react with BChE (Figure S13, Supporting Information). The blood samples were analyzed by the combination of those probes, and varied activity clusters with different activity preferences toward ATC and HCCA‐Ac were detected (Figure 3G,H; Figure S13, Supporting Information). With the comparison with the recombinant enzymes, the enzyme that showed fluorescent signals mainly from ATC and not from HCCA‐Ac (cluster II in Figure 3H) were assigned as BChE. Activity spots that corresponded with recombinant AChE (cluster V in Figure 3H) was hardly detectable in blood, presumably reflecting the low concentration of AChE. Another major enzyme species (I and IV in Figure 3H) showed good reactivities toward both ATC and HCCA‐Ac. They did not seem to exhibit the strict substrate recognition and were considered as general esterase species such as CES1, CES2, and CES3.^[^ ^30^, ^31^ ^]^ Currently, alterations of blood ChE activities are studied in various health conditions,^[^ ^9^ ^]^ but our observation implied that the result of conventional bulk assay looks at the mixture of diverse enzyme species, which can pose a risk of losing the information from activity alterations of minor enzyme species. Then, we have tested if our single‐molecule esterase assay platform can detect the biomarker enzymes of liver damage in blood samples, using thioacetamide (TAA)‐treated mice as acute liver damage model (Figure S14, Supporting Information).^[^ ^32^ ^]^ In single‐molecule esterase assays, there was a decrease of the number of molecules of one of the ChE species (cluster II), which was assigned as BChE (Figure 3I; Figure S12, Supporting Information). BChE was synthesized in the liver, and this result was consistent with the current understanding that the malfunction of liver leads to the decreased production of BChE.^[^ ^33^ ^]^ Another notable change was that the enzyme species with very weak reactivity toward HCCA‐Ac (cluster III) increased upon liver damage (Figure S15, Supporting Information). These changes were not detectable using conventional 384‐well plate‐based fluorometric assays performed in bulk conditions (Figure S14, Supporting Information), and the overall results show that the single‐molecule enzyme activity assay developed in this study can offer practical information for activity‐based diagnosis, with the ability of detecting diverse single‐molecule enzyme species simultaneously in blood samples.

## 3 Conclusion

In this study, we have developed the system of coupled thiol‐forming assay‐based detection of enzyme activities at single‐molecule level that widen the scope of enzymes monitorable in this informative biochemical assay platform. The key to the design was the control of the hydrophilicity of the probes, and newly developed boron dipyrromethene fluorophore with phosphonate substitution served best for the purpose, by showing a superior hydrophilicity than other presently available water‐soluble BODIPY derivatives. We have utilized the established assay to detect the single‐molecule activities of ChEs and have shown that they were detectable in blood samples and can be used as a source of activity‐based diagnosis. The focus of the present study was on ChEs, while diverse enzymes that generates thiols from natural thiol‐containing metabolites or synthetic thioesters can be targetable in the same concept, so it can serve as a useful platform to study various enzyme activities at proteoform levels.

## 4 Experimental Section

### Materials

Reagents and solvents were of the best grade available, supplied by Tokyo Chemical Industries, FUJIFILM Wako Pure Chemical Corporation, Sigma‐Aldrich, Dojindo, Kanto Chemical Co., Watanabe Chemical Industries, and were used without further purification. Enzymes were purchased from Sigma‐Aldrich and was used without further purification.

### Instruments

NMR spectra were recorded on a JEOL JNM‐LA400 instrument at 400 MHz for ^1^H NMR and at 100 MHz for ^13^C NMR. Mass spectra (MS) were measured with a JEOL JMS‐T100LC AccuToF (ESI). Preparative HPLC was performed on an Inertsil ODS‐3 (10.0 × 250 mm) column (GL Sciences Inc.) using an HPLC system composed of a pump (PU‐2080, JASCO) and a detector (MD‐2015 or FP‐2025, JASCO). LC‐MS analyses were performed on a Waters Acquity UPLC (H class)/QDa quadrupole MS analyzer or Acquity UPLC (H class)/Xevo TQD quadrupole MS/MS analyzer equipped with an Acquity UPLC BEH C18 column (Waters). Column chromatography using silica gel was performed on a MPLC system (Yamazen Smart Flash EPCLC AI‐5805 (Tokyo, Japan)). Reversed‐phase MPLC purification was performed on an Isolera One (Biotage) equipped with a SNAP Ultra C18 30 g (Biotage).

### UV–vis Absorption and Fluorescence Spectroscopy

UV‐Visible spectra were obtained on a Shimadzu UV‐1800. Fluorescence spectroscopic studies were performed on a Hitachi F7000. The slit width was 5 nm for both excitation and emission. The photomultiplier voltage was 400 V.

### Computational Details

Computational calculations were performed using the Gaussian16 program. For the calculation of the solvation free energy, the entire structure of the molecule was calculated. Geometry optimization and vibrational analysis of each compound were performed at the B3LYP/6‐31+G(d,p) level including water/diethyl ether in the SMD solvent model. Stationary points were optimized without any symmetry assumptions. For the calculation of HOMO energy, only the BODIPY moiety without ß‐Nitrostyrene was calculated. The calculation was performed at B3LYP+D3/6‐311++G(2d,p)//B3LYP/6‐31+G(d,p) level including water in the SMD solvent model. Stationary points were optimized without any symmetry assumptions.

### Digital Enzyme Assay in Microdevice

Digital enzyme assays were performed using microdevices prepared according to the literature^[^ ^8^ ^]^ or ones commercially available.^[^ ^34^ ^]^ For preparation of the device, a glass coverslip (24 × 32 mm) was immersed in 8 m KOH, sonicated for 90 min, and incubated overnight. The coverslip was then rinsed with pure water and dried using an air blowing gun. It was spin‐coated with an amorphous fluorocarbon polymer (9% CYTOP; AGC, Japan) at 1000 rpm for 30 s, baked at 80°C for 10 min, and then heated at 180°C for 1 h on a hotplate. The thickness of the CYTOP layer is 1.6 µm. A CYTOP‐coated coverslip was spin‐coated with a positive photoresist (AZ P4620; AZ Electronic Materials, USA) at 7500 rpm for 30 s and baked at 100°C for 5 min. After rehydration of the photoresist at 25°C under 60% humidity, photolithography was carried out using a photomask with 1.8 µm holes, separated by 8 mm, and then incubated for 90 s in a developer (AZ300 MIF, AZ Electronic Materials, USA). The resist‐patterned coverslip was dry‐etched with O_2_ plasma using a reactive ion etching system (DES‐101E; YAC, Japan or RIE‐10NR or PC‐300; Samco, Japan) to remove the exposed CYTOP. The substrate was cleaned and rinsed with acetone, isopropanol, and pure water to remove any remaining photoresist. The resulting CYTOP‐on‐coverslip substrate contained an array of exposed SiO_2_ patterns with diameter of 3.2 µm. For the loading of the sample, 15 µL mixture of enzyme and reagents in buffer (HEPES buffer (100 mm, pH 7.4) containing Triton X‐100 (3 mm)) was loaded into the loading port of the device, and put on ice for 5 s. Then, the chambers were sealed FC‐40 (30 µL) was gradually introduced and exchanged with Fomblin PFPE (40 µL). For the imaging with the commercially available device (Simoa disk; Quanterix), 40 µL of r mixture of enzyme and reagents in buffer was loaded into microdevice by manual pipetting. Then, 80 µL of FC‐70 (Sigma‐Aldrich) was introduced into the device to flush out an excess amount of the reaction mixture. The enzymatic activity in the chambers was measured using an epifluorescence microscope or confocal fluorescence microscope.

### Epifluorescence Microscopy

Fluorescence images were acquired by microscope (Ti2, Nikon) equipped with a 20× dry objective lens (Plan Apo VC 20×), sCMOS camera (ORCA‐Fusion C14440, Hamamatsu Photonics), white LED illumination unit (X‐Cite Xylis, Opto Science), and a motorized stage. The assay was performed using a solution containing sTM^[^ ^6^ ^]^ (10 µM) as the internal standard, and the focus was adjusted using its fluorescence. Images were acquired in tile scan mode with perfect focus. The excitation and emission filters used were FITC (mirror = 510 nm, Ex. = 460–500 nm, Em. = 510–560 nm) and mCherry (mirror = 600 nm, Ex. = 550–590 nm, Em. = 608–683 nm), respectively.

### Confocal Microscopy

Fluorescence images were acquired by confocal fluorescence microscope unit (AX, Nikon) equipped with a 20× dry objective lens (Plan Apo VC 20×), LASER unit (LUA‐S4), detector unit (DUX‐ST), and a motorized stage. The assay was performed using a solution containing sTM^[^ ^6^ ^]^ (10 µM) as the internal standard, and the focus was adjusted using its fluorescence. Images were acquired in tile scan mode with perfect focus. The excitation wavelength and emission filters used were DAPI (Ex. = 405 nm, Em. = 429–474 nm), FITC (Ex. = 488 nm, Em. = 500–550 nm) and Cy3 (Ex. = 561 nm, Em. = 570–616 nm), respectively. Images were taken as sequential acquisition mode.

### Image Processing

Images were processed using the GA3 module of NIS Elements software (Nikon). First, all fluorescence images were background‐corrected using a rolling ball correction (3 µm). Then, ROIs were chosen by bright spot detection at mCherry filters (diameter = 3 µm), and irregular fluorescent spots derived from fluorescent debris or air bubbles were omitted by dilating the ROI and removing the overlapping ROIs. For the counting of the active enzyme spots, the spots were chosen by bright spot detection at FITC filters. The fluorescence signals were acquired as the mean of the signals from the nine pixels at the center of each ROI. Data were processed using Excel or Kaleidagraph software to construct histograms and scatter plots.

### n‐Octanol‐Water Separation

Probes (10 µm) were dissolved in the mixture of n‐Octanol (30 µL) and PBS (pH 7.4, 100 mm, 30 µL) in 1.5 mL plastic tube and vortexed at 25˚C for 30 min. The 10 µL of organic layer and aqueous layer were collected and was mixed with 30 µL DMSO. The collected solutions were analyzed by LC‐MS/MS using multiple reaction monitoring (MRM) mode. *K*_OW_ values were calculated by dividing the amount of probe detected in organic layers by that in aqueous layer.

### Enzyme Activity Assay using Microplate Reader

Enzyme assay was performed in phosphate‐buffered saline (pH 7.4). Half‐area 384‐well plates (Corning 3677) were used for the assay (20 µL reaction volume). Fluorescence was detected with a plate reader, EnVision 2103 Multilabel Reader (Perkin Elmer), with appropriate filter settings.

### Plasma Samples from Healthy Human Subjects

Plasma samples from healthy human subjects were collected from Kagoshima Prefectural Comprehensive Health Center with the Program for Promotion of Fundamental Studies in Health Sciences conducted by the National Institute of Biomedical Innovation of Japan, Health and Labour Sciences Research Grants from the Ministry of Health, Labor and Welfare of Japan, and P‐CREATE of the Japan Agency for Medical Research and Development (AMED). Ethical approval for this study was obtained from the central ethics committees of Nippon Medical School (M‐2021‐002) and the ethical committee of Nippon Medical School (A‐2020‐032 and A‐2020‐044).

### Plasma Samples from Mice

Ethical approval for the study using animals was obtained from Animal Care and Use Committee of The University of Tokyo (P4‐21, P31‐9). Six‐week‐old male C57BL/6JJcl mice were purchased from CLEA Japan (Tokyo, Japan) and acclimatized for five days. The mice were exposed to thioacetamide (TAA, T0817, Tokyo Chemical Industry Co., Japan) dissolved in drinking water (300 mg L^−1^) to induce liver damage, whereas the controls received a tap water. After four days of treatment, mice were euthanized and the blood was collected through an inferior vena cava into 1.5 mL tube containing 1.5 µL heparin (Yoshindo Inc, Japan). The collected blood sample was centrifuged (1700 g, 4°C for 15 min) for plasma separation. Plasma alanine aminotransferase (ALT) and aspartate aminotransferase (AST) were measured using a DRI‐CHEM NX500sV (Fujifilm Corporation, Japan).

### Statistical Analysis

For the data that involved statistical analysis, the experiments were performed with indicated replicates (n), and the data is presented as mean ± S. D. (error bars). For Figure 3D,E, the signal was normalized to the average fluorescence intensities of blank wells. Statistical analysis was performed with Student's *t*‐test (two‐sided test, equal error variances) using Microsoft Excel.

## Conflict of Interest

Shingo Sakamoto is cofounder, employee, and shareholder of Cosomil, Inc. Toru Komatsu, Tadahaya Mizuno, Rikiya Watanabe, and Kazufumi Honda are advisor and shareholder of Cosomil, Inc. Tatsuya Ukegawa, Toru Komatsu, Shingo Sakamoto and Yasuteru Urano are inventors of a patent of the probes.

## Supporting information

Supporting Information

## Data Availability

The data that support the findings of this study are available from the corresponding author upon reasonable request.
