# Supplementary material for: Thioester‐Based Coupled Fluorogenic Assays in Microdevice for the Detection of Single‐Molecule Enzyme Activities of Esterases with Specified Substrate Recognition
Source: Adv Sci (Weinh). 2023 Dec 22;11(10):2306559. doi: 10.1002/advs.202306559 (PMC10933651; doi:10.1002/advs.202306559)
Supplement: Supplementary file 1 — Supporting Information [file ADVS-11-2306559-s001.pdf]

ADVANCED SCIENCE
Open Access

Supporting Information

for *Adv. Sci.*, DOI 10.1002/advs.202306559

Thioester-Based Coupled Fluorogenic Assays in Microdevice for the Detection of Single-Molecule Enzyme Activities of Esterases with Specified Substrate Recognition

Tatsuya Ukegawa, Toru Komatsu, Mayano Minoda, Takuya Matsumoto, Takumi Iwasaka, Tadahaya Mizuno, Ryo Tachibana, Shingo Sakamoto, Kenjiro Hanaoka, Hiroyuki Kusuhara, Kazufumi Honda, Rikiya Watanabe and Yasuteru Urano**

Supporting Information

Thioester-based coupled fluorogenic assays in microdevice for the detection of single-molecule enzyme activities of esterases with specified substrate recognition

Tatsuya Ukegawa, Toru Komatsu, Mayano Minoda, Takuya Matsumoto, Takumi Iwasaka, Tadahaya Mizuno, Ryo Tachibana, Shingo Sakamoto, Kenjiro Hanaoka, Hiroyuki Kusuhara, Kazufumi Honda, Rikiya Watanabe, Yasuteru Urano**

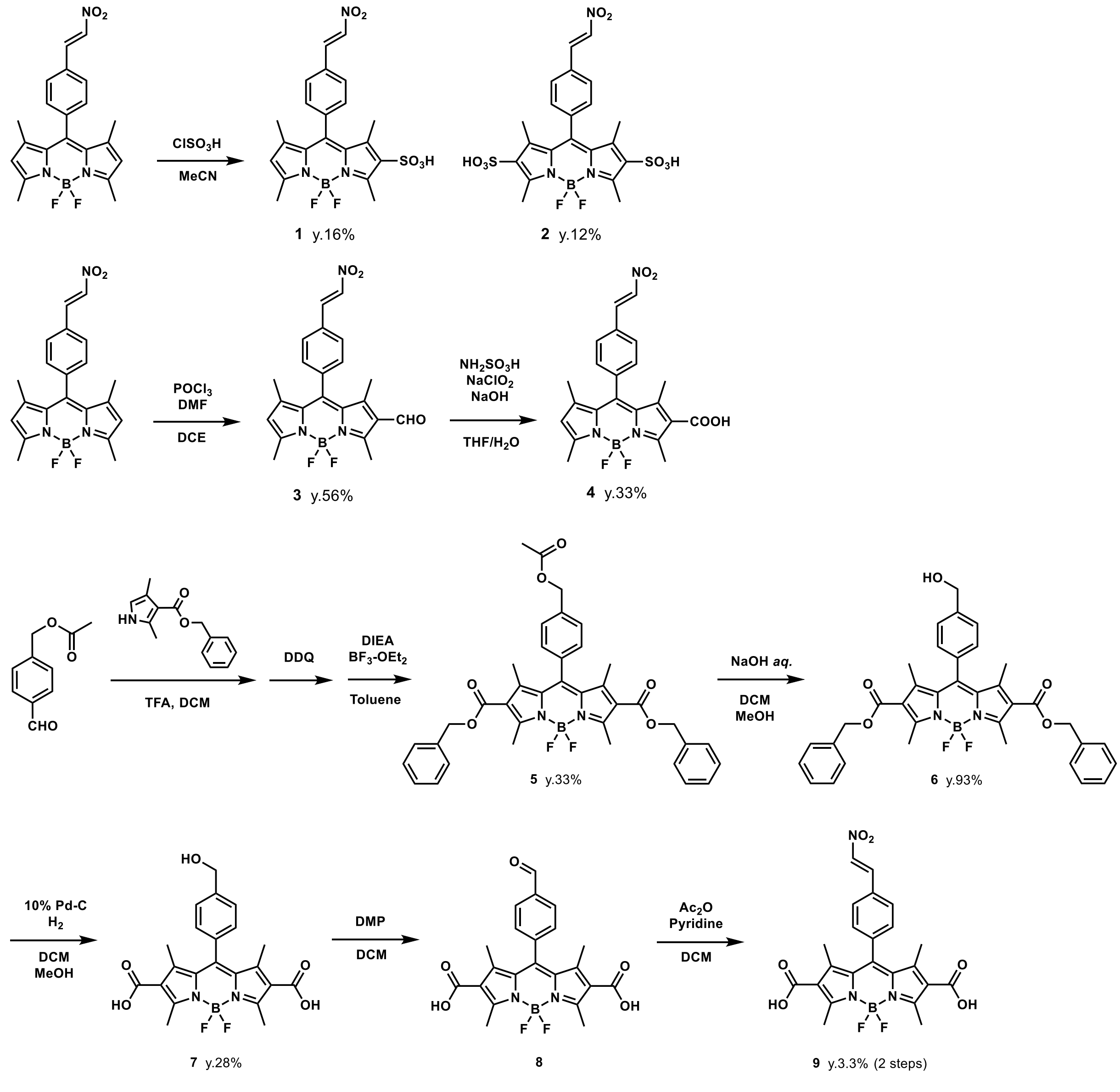

Scheme S1. Synthetic schemes of sulfonated and carboxylated 8-(*p*-nitroolefin)-BODIPY

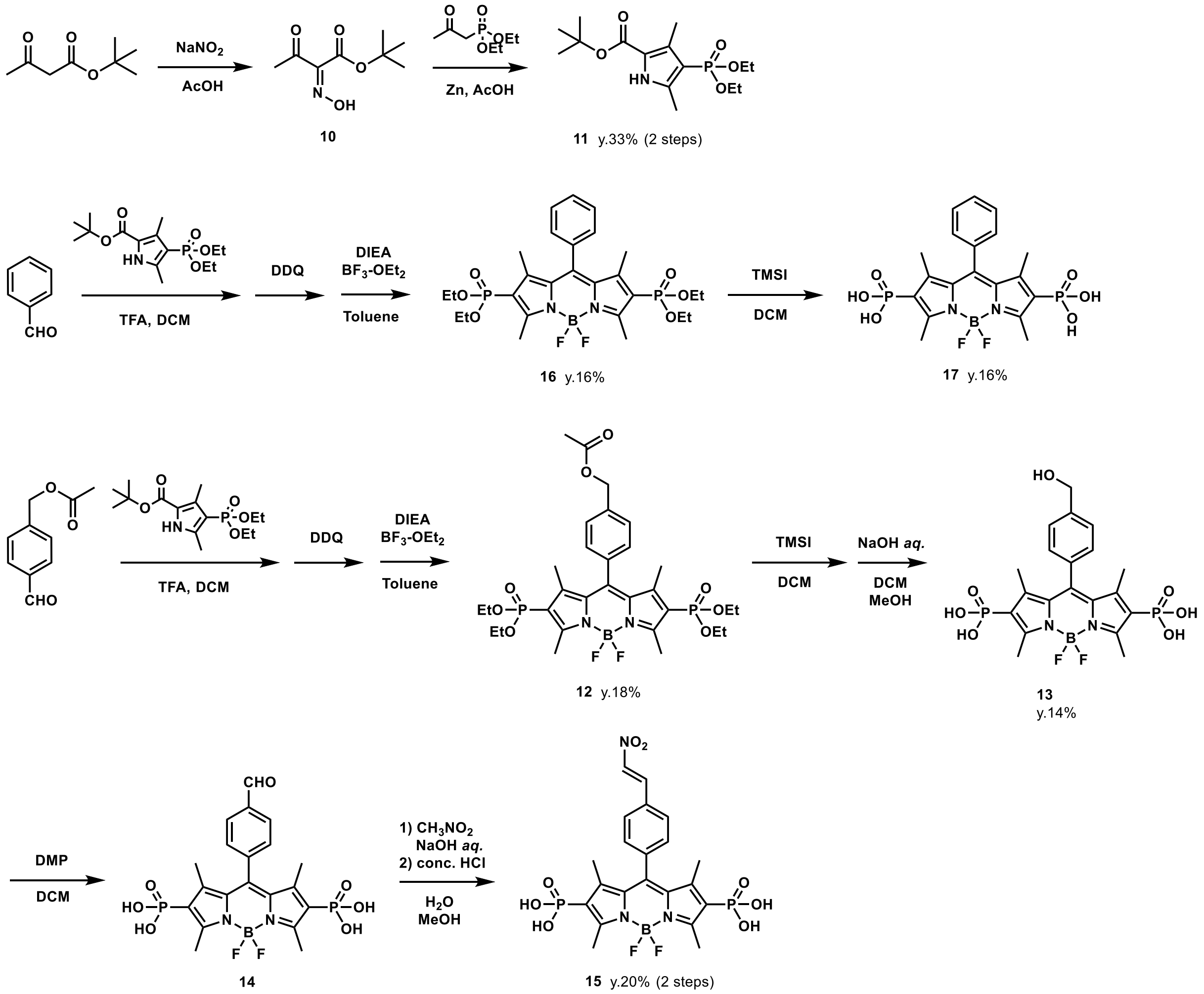

Scheme S2. Synthetic schemes of dpNOB.

Table S1. Expected concentrations of fluorogenic products in microfabricated chamber in single-molecule enzyme activity assay. The calculation was performed with enzymes with varied turnover number k_{cat}, chamber volume = 50 fL and under the assumption that the formation of fluorescent product is linear.

Time	1 min	1 h	1 d
k_{cat} = 13.7 (sec^{-1}) (median of values found in BRENDA)[35]	0.08 µM	4.8 µM	116 µM
k_{cat} = 336 (sec^{-1}) (BChE)[27]	2.0 µM	118 µM	
k_{cat} = 6,500 (sec^{-1}) (AChE)[26]	38 µM	2287 µM	

Table S2. Relationship between the number of active enzyme spots in single-molecule enzyme activity assays and concentration of the active enzyme. Calculation was performed with chamber volume = 50 fL, number of chambers = 160,000, and MW = 440 kDa (BChE) and under the assumption of the even distribution of enzymes into the chamber.

Spot number (out of 160,000 chambers)	10	100	1,000
Concentration	2.1 fM<br>0.91 pg/mL	20.8 fM<br>9.1 pg/mL	208 fM<br>91 pg/mL

Table S3. Computational calculations used to design the performances of nitroolefin-based probes.

Compounds	R^2/R^6	Charge	HOMO energy level (Hartree)[1)]	Solvation free energy (water - gas phase, kcal/mol)[2)]	Solvation free energy (water - Et_2O, kcal/mol)[2)]
NOB	H/H	Neutral	-0.2084	-13.5680	8.7632
cNOB	H/CO_2^-	Monoanion	-0.2077	-65.4468	-14.3599
sNOB	H/SO_3^-	Monoanion	-0.2129	-65.2679	-14.0663
dcNOB	CO_2^-/CO_2^-	Dianion	-0.2073	-157.7736	-46.5951
dsNOB	SO_3^-/SO_3^-	Dianion	-0.2165	-154.4246	-44.2144
dpNOB (dianionic)	PO_3H^-/PO_3H^-	Dianion	-0.2128	-157.0921	-47.8671
dpNOB (tetraanionic)	PO_3^{2-}/PO_3^{2-}	Tetraanion	-0.1983	-523.5768	-174.0430

1) Calculation was performed with BODIPY without substitution at 8th position.
2) Calculation was performed with BODIPY with 8-nitroolefin substitution.

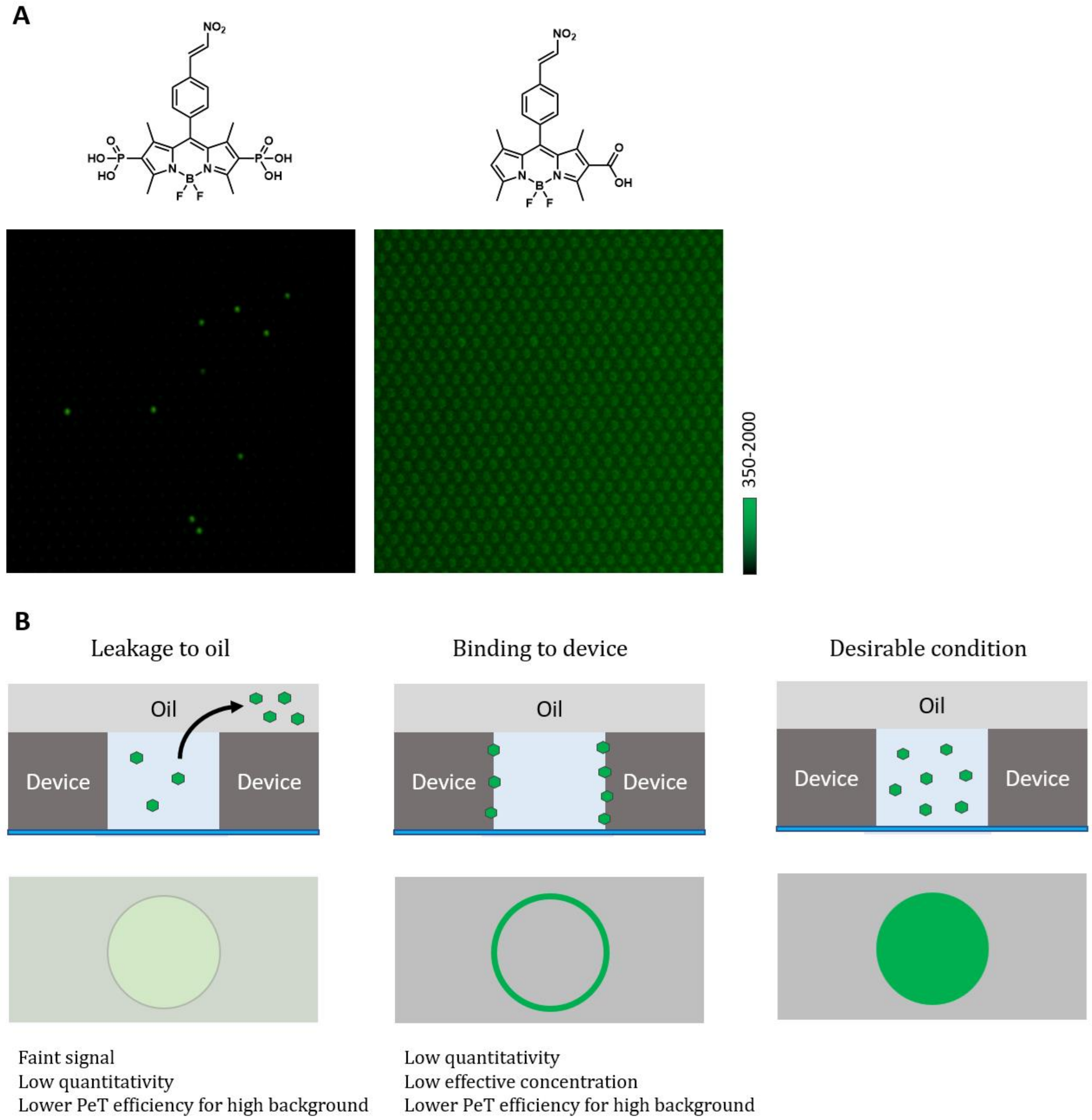

Figure S1. Importance of high hydrophilicity of sensors for microdevice-based single-molecule enzyme activity assays. (A) Epifluorescence images of microdevice loaded with recombinant BChE (from equine serum, 0.1 ng/mL) with dpNOB (left) or cNOB (right, 30 μM) and acetylthiocholine iodide (ATC, 1 mM) in HEPES Buffer (10 mM, pH 7.4, containing 0.1% CHAPS) and incubated at 25˚C for 30 min. (B) Possible negative effects (leakage to oil or binding to device) occurring in single-molecule enzyme assays as a result of insufficient hydrophilicity of the probes.

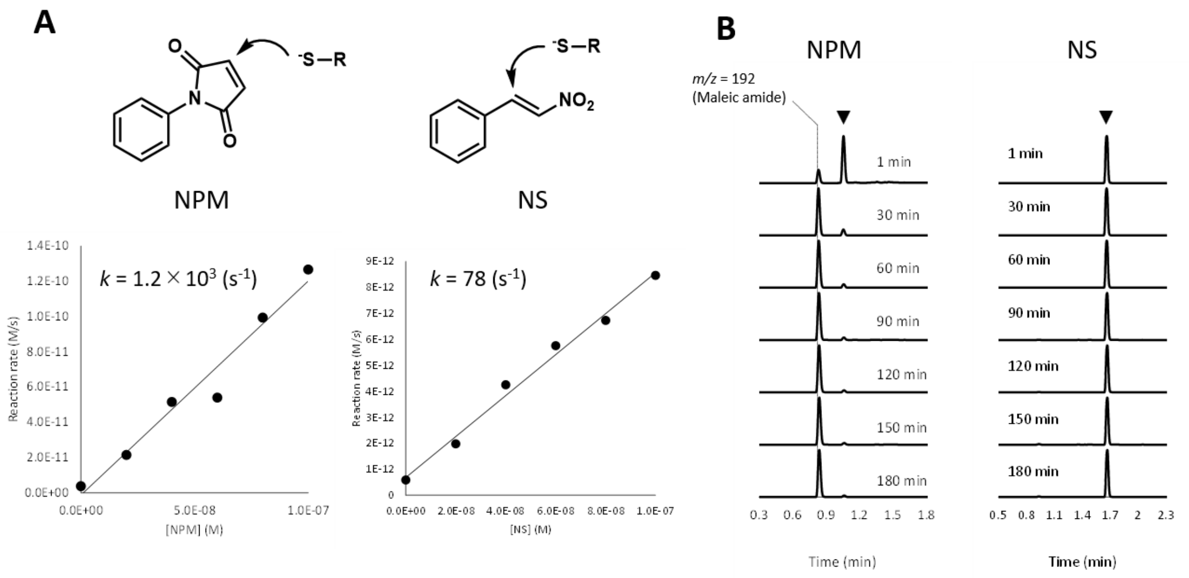

Figure S2. Reactivity and stability of *N*-phenylmaleimide (NPM) and nitrostyrene (NS). (A) NPM and NS (0-100 nM) were reacted with glutathione (1 mM) in PBS (pH 7.4) for 3 min and the initial reaction rate was calculated by detecting the NPM-glutathione adduct and NS-glutathione adduct in LC-MS/MS analysis. *k* was calculated based on pseudo first-order kinetics (Ostwald isolation). (B) LC-MS chromatogram (280 nm absorbance) of NPM and NS (10 μM) in PBS (pH 7.4) at 25°C for 1-180 min. The peak observed at 0.82 min in NPM exhibited the *m/z* value of 192 (ESI^+), indicating the formation of maleic amide, a hydrolysis product of imide.

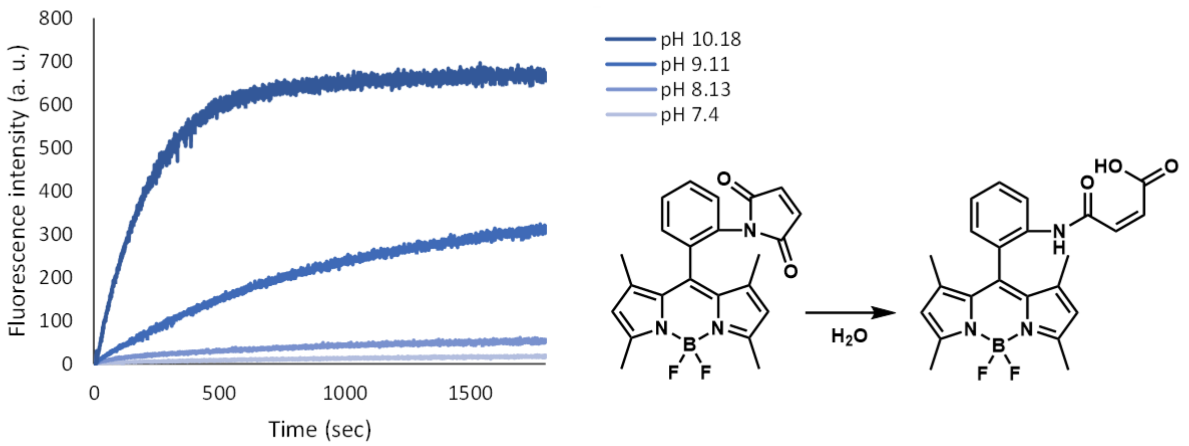

Figure S3. Instability of NPM-based probes in aqueous media. Fluorescence change of *N*-phenylmalemide-substituted-BODIPY[12] (1 μM) in sodium phosphate buffer (100 μM) with various pH.

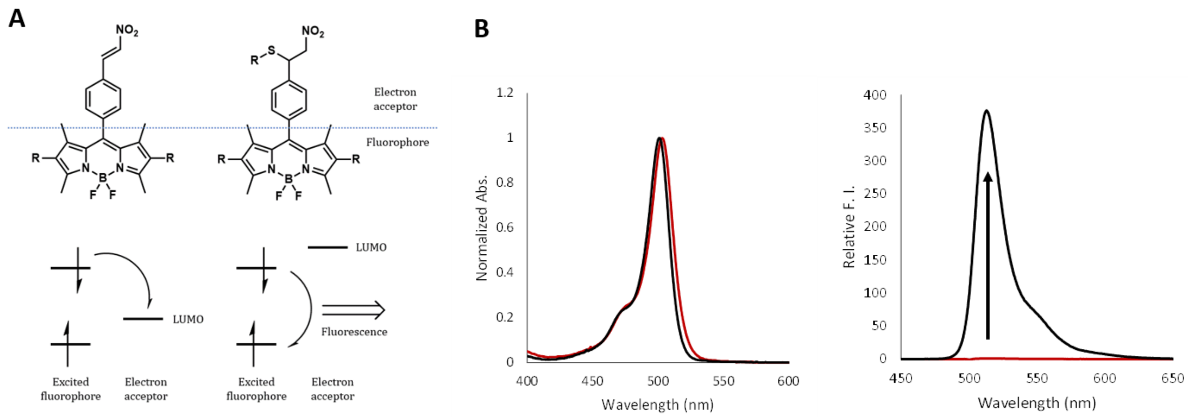

Figure S4. Design of fluorescence activation of nitroolefin-BODIPY by d-PeT mechanism. (A) Design of d-PeT-based fluorescence control of probes. (B) Absorbance and fluorescence spectra of nitroolefin-BODIPY (1 μM) before (red) and after (black) reacting with *N*-acetylcysteine (100 μM) in phosphate buffer (100 mM, pH 7.4) containing 33% DMSO as a cosolvent.

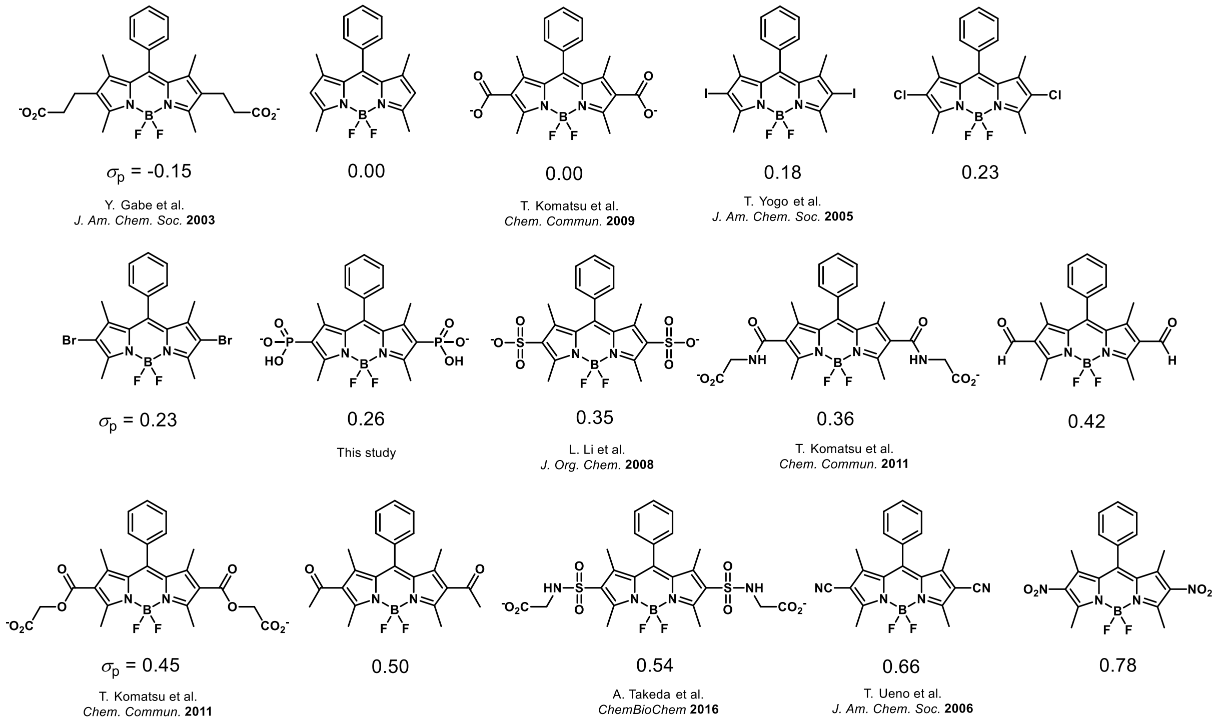

Figure S5. List of 2,6-substituted BODIPY derivatives that exhibit fluorescence. σ_p is Hammett constant of the substituents[20].

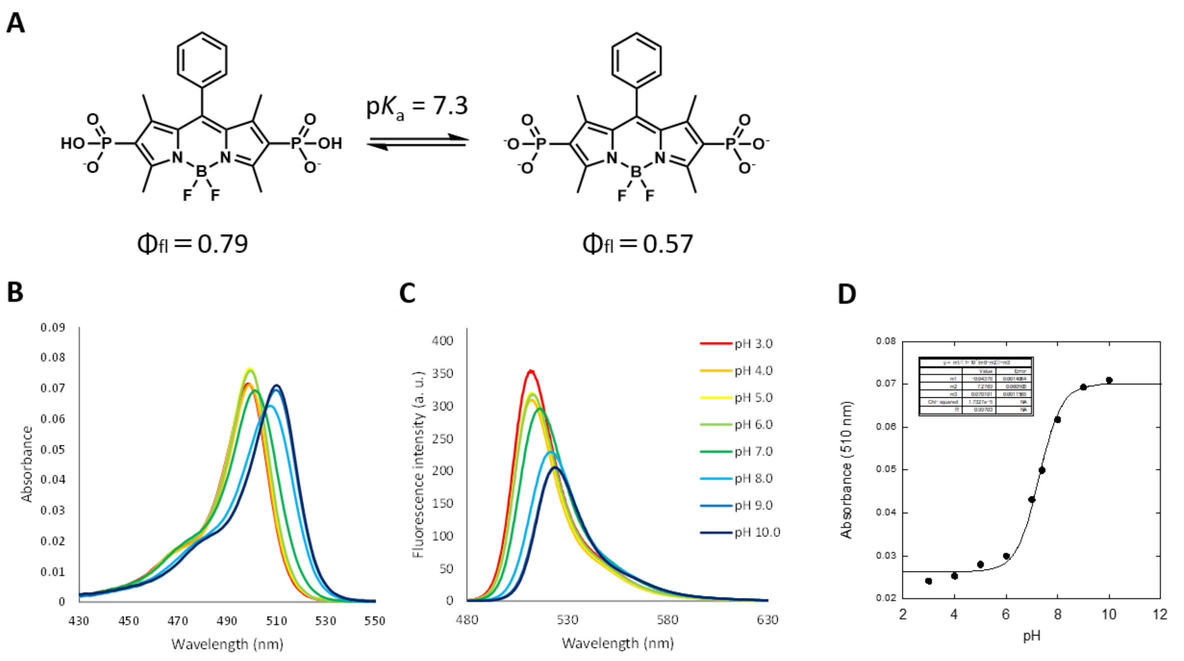

Figure S6. Absorbance/fluorescence characteristics of dpBODIPY. (A) Expected equilibrium of dpBODIPY and quantum yields measured in pH conditions in which the indicated form is considered major (pH 5 for PO_3H^- form and pH 9 for PO_3^{2-} form). (B) Absorbance spectra of dpBODIPY (1 μM) at sodium phosphate buffer (100 mM) with varied pH. (C) Fluorescence spectra of pBODIPY (1 μM) at sodium phosphate buffer (100 mM) with varied pH. (D) pH-dependent curve of 510 nm absorbance and fitting to calculate apparent pK_a.

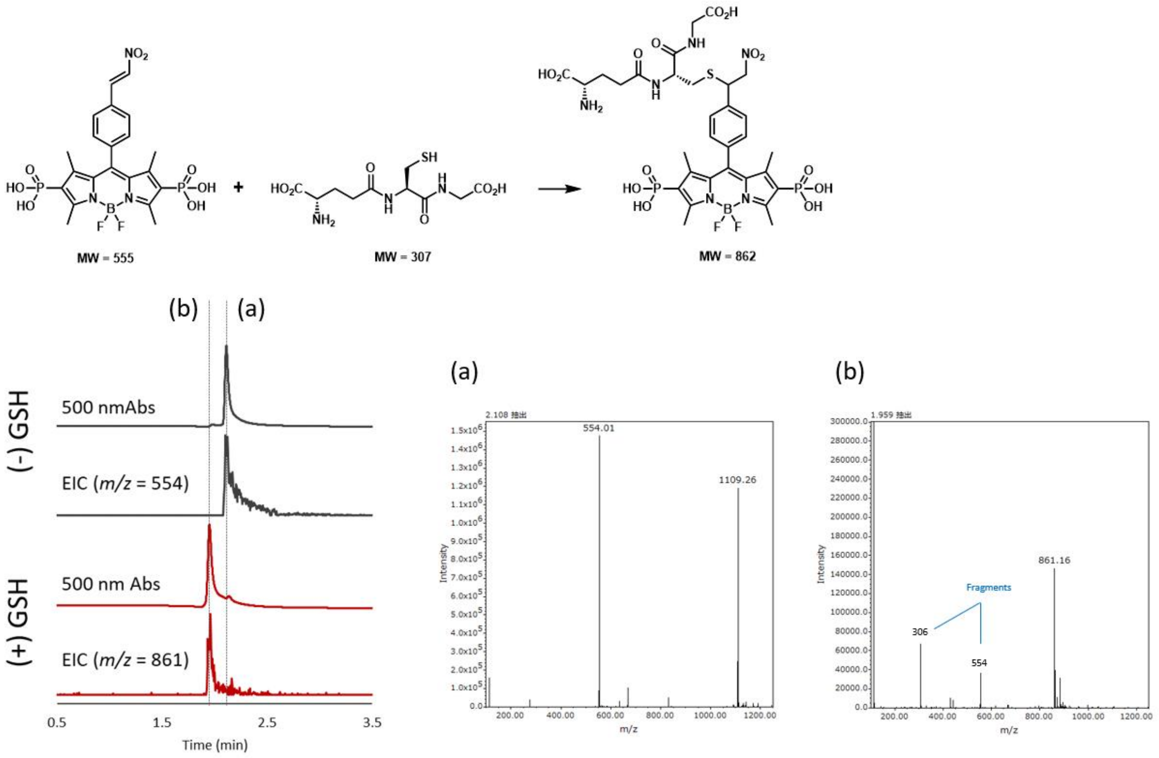

Figure S7. Confirmation of reaction of dpNOB with GSH using LC-MS. dpNOB (10 μM) mixed with or without GSH (30 μM) in PBS (pH 7.4) and incubated at 25˚C for 30 min was analyzed by LC-MS. (Left) 500 nm absorbance chromatograms (top) and extract ion chromatograms (EIC, bottom) of dpNOB incubated with or without GSH. (Right) Mass spectra observed in LC-MS-based analysis of the reaction at time (a) and (b).

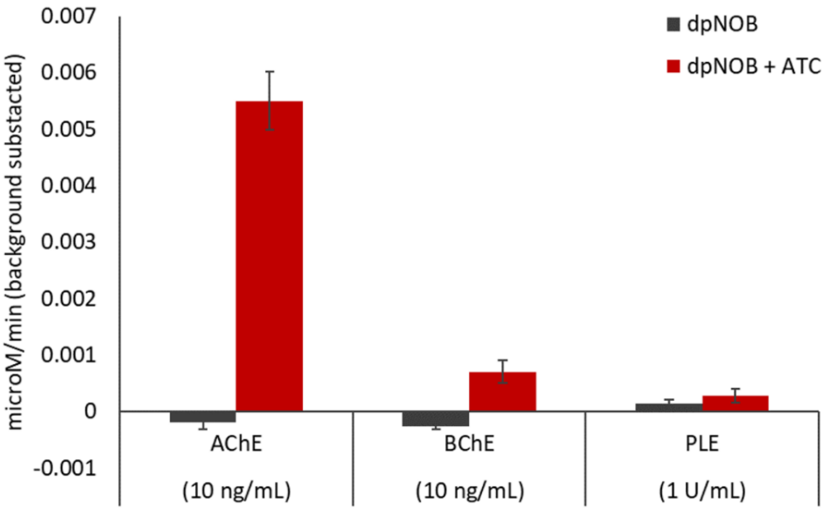

Figure S8. Reactivity of ATC against recombinant enzymes. dpNOB (1 µM) was mixed with or without acetylthiocholine iodide (100 µM) and enzymes in PBS (pH 7.4) containing 0.1% CHAPS and incubated at 25°C. Initial fluorescence increase rate at 5-30 min was monitored and converted to concentration changes using dpBODIPY (1 µM) as a standard. Ex/Em = 478-492 nm/523-548 nm. Error bars represent S. D. (n = 3).

A

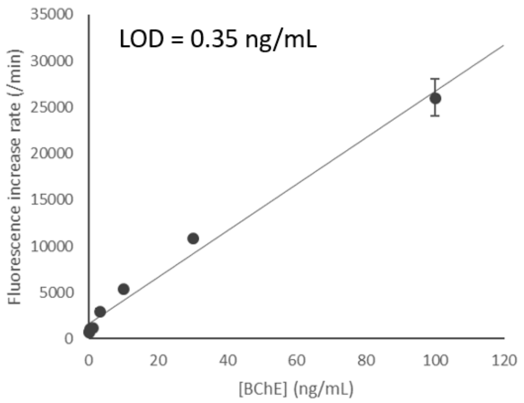

B

AChE (Sigma-Aldrich C2888)
200-1,000 units/mg protein
(1 unit hydrolyze 1 μmol acetylcholine per min)
60 ± 6 nmol ATC/min/ng prot.

BChE (Sigma-Aldrich C7512)
14 units/mg protein
(1 unit hydrolyze 1 μmol butyrylcholine per min)
2.0 ± 0.3 nmol ATC/min/ng prot.

Figure S9. Activities of AChE and BChE studied in conventional 384-well plate-based assay. dpNOB (10 μM) was mixed with acetylthiocholine iodide (ATC, 500 μM) and varied concentrations of BChE in PBS (pH 7.4) containing 0.1% CHAPS and incubated at 25°C. Initial fluorescence increase rate at 5-30 min was monitored and converted to concentration changes using the calibration curve prepared by detection of GSH in the same condition. (A) Calibration curve of concentration of BChE and the fluorescence increase rate (/min). Error bars represent S. D. (n = 4). Limit of detection (LOD) was calculated as the value corresponding to 3.29 σ. (B) Expected unit number and the observed activities toward acetylthiocholine (ATC) of AChE and BChE. The values were calculated from the activities measured with 1 ng/mL enzymes and shown as mean ± S. D. (n = 4).

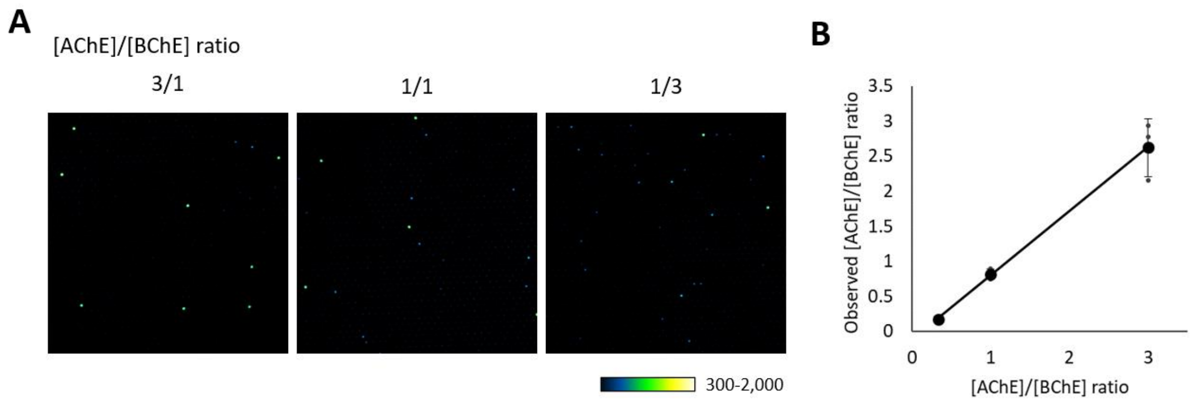

Figure S10. Discrimination of different ChE species in single-molecule enzyme activity analysis. (A) Epifluorescence images of microdevice containing mixture of AChE and BChE (AChE 1 ng/mL/BChE 0.33 ng/mL ([AChE]/[BChE] ratio = 3/1), AChE 1 ng/mL/BChE 1 ng/mL (1/1), and AChE 0.33 ng/mL/BChE 1 ng/mL (1/3)) with fluorescence probes (30 μM) with or without ATC (1 mM) in HEPES Buffer (10 mM, pH 7.4, containing 0.1% CHAPS), after incubation at 25˚C. (B) Observed numbers of spots of [AChE] and [BChE] ([AChE]/[BChE] ratio, vertical axis) was plotted against the expected ratio (horizontal axis). Fluorescence spots were counted in the images acquired at 1 min and 5 min (contrast > 400), and the spots that were detected at both timepoints were identified as [AChE] and those detected only at 5 min were identified as [BChE]. Error bars represent S. D. (n = 3).

A

Sample	[BChE]	[GSH]
**a**	10 ng/mL	0 μM
**b**		0.1 μM
**c**		0.3 μM
**d**		1 μM
**e**		3 μM
**f**		10 μM

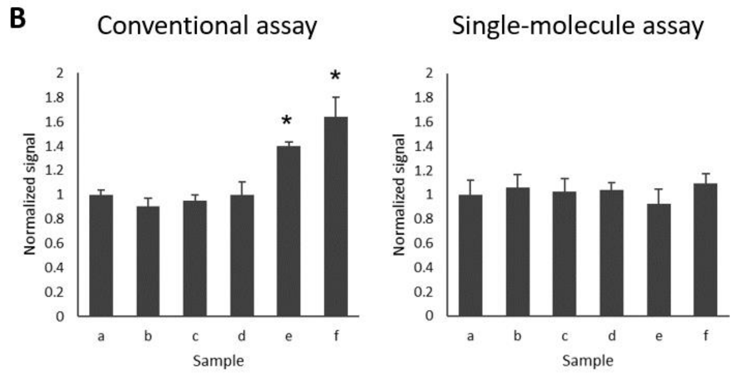

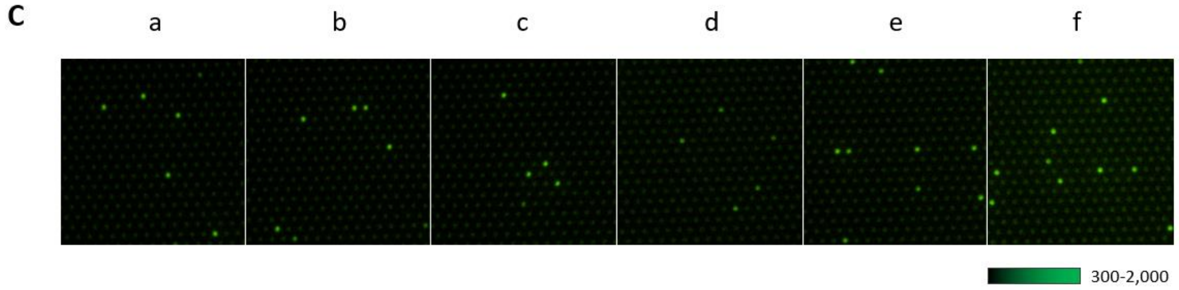

Figure S11. Detection of butyrylcholinesterase (BChE) activities in the sample containing varied concentrations of thiols. (A) Preparation of samples containing BChE and varied concentrations of glutathione (GSH). (B) (left) Detection of BChE activities using conventional 384-well plate-based assay. The assay was performed by incubating dpNOB (10 μM) with acetylthiocholine iodide (500 μM) and sample a-f (BChE concentrations were set to 10 ng/mL) in PBS (pH 7.4) containing 0.1% CHAPS and incubated at 25°C for 30 min. The signals (fluorescence increase rate over 30 min) were normalized to that of sample a (0 μM GSH). Error bars represent S. D. (n = 4). *$P < 0.05$ (Student's t-test). (right) Detection of BChE activities using single-molecule enzyme activity assay. The assay was performed by loading dpNOB (30 μM) with ATC (500 μM) and sample a-f (BChE concentrations were set to 0.1 ng/mL) in HEPES Buffer (10 mM, pH 7.4, containing 0.1% CHAPS) into microdevice and incubating after incubation at 25˚C for 30 min. The signals (count of activity spots) were normalized to that of sample a (0 μM GSH). Error bars represent S. D. (n = 4). (C) Epifluorescence images of samples a-f in (B).

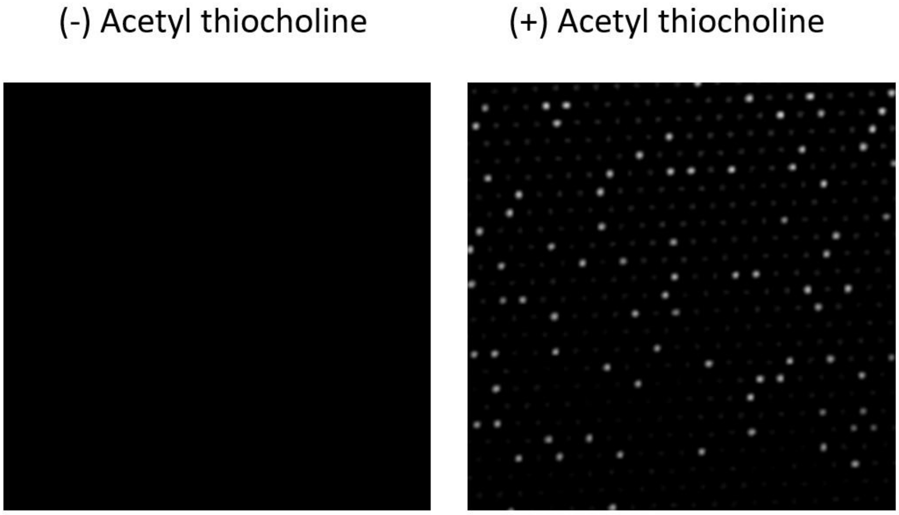

Figure S12. Dependency of fluorescence signal generation in blood on ATC. Epifluorescence images of microdevice containing human plasma (3000-fold dilution) with fluorescence probes (30 μM) with or without ATC (1 mM) in HEPES Buffer (10 mM, pH 7.4, containing 0.1% CHAPS), after incubation for 30 min at 25˚C.

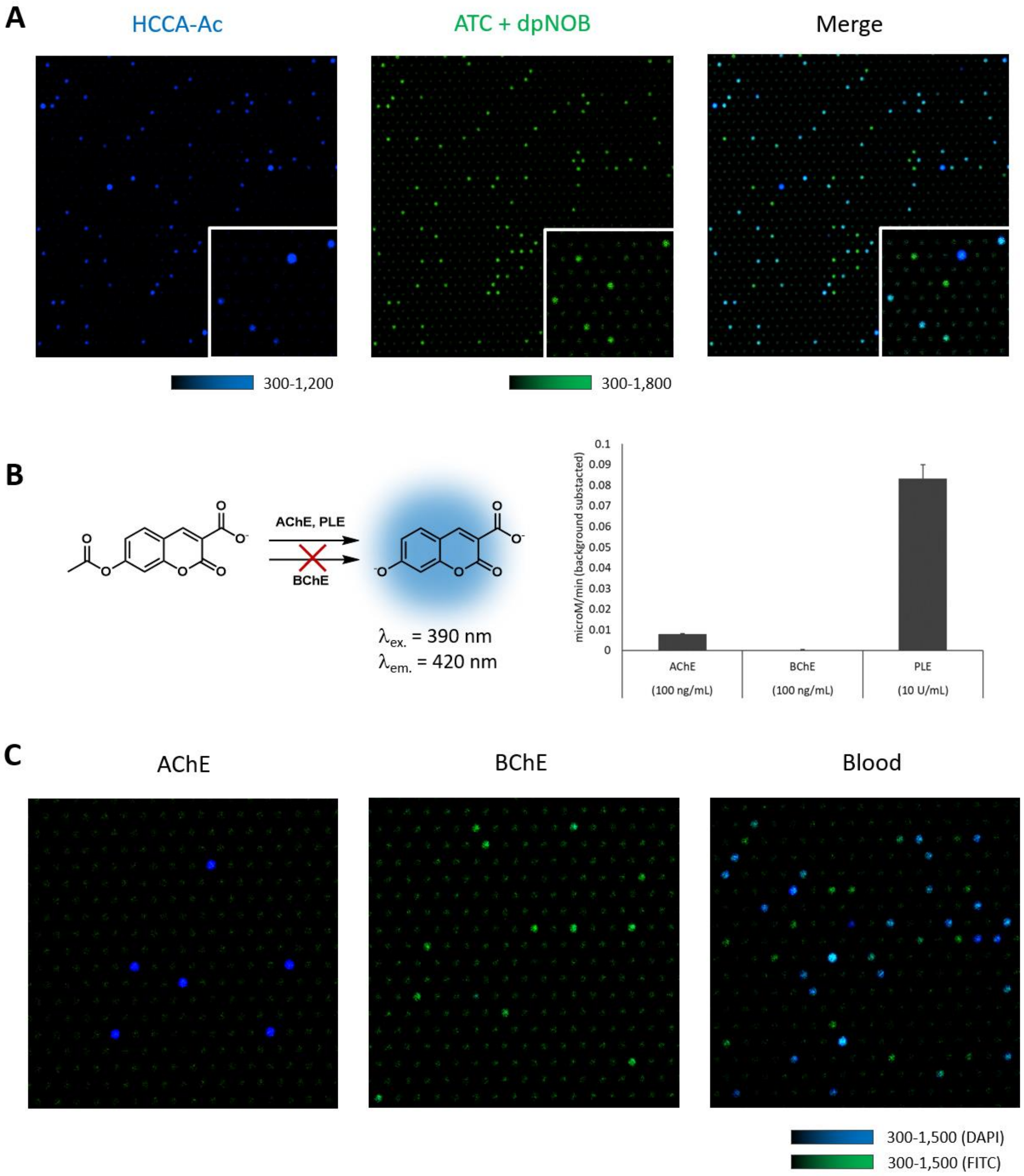

Figure S13. Detection of various esterase species in blood samples. (A) Confocal fluorescence images of microdevice loaded with 1/3000 diluted human plasma samples mixed with ATC (1 mM), dpNOB (30 μM), and HCCA-Ac (100 μM) and incubated for 2 h. (B) Fluorescence intensity of HCCA-Ac (10 μM)[29] after mixed with enzymes in PBS (pH 7.4) containing 0.1% CHAPS and incubated at 25°C. Initial fluorescence increase rate at 5-30 min was monitored and converted to concentration changes using HCCA (10 μM) as a standard. Ex/Em = 335-375 nm/448-473 nm. Error bars represent S. D. (n = 3). (C) Overlayed confocal fluorescence images of microdevice loaded with recombinant AChE (1 ng/mL) BChE (100 ng/mL) or 1/3000 diluted human plasma samples mixed with ATC (1 mM), dpNOB (30 μM), and HCCA-Ac (100 μM) and incubated for 2 h.

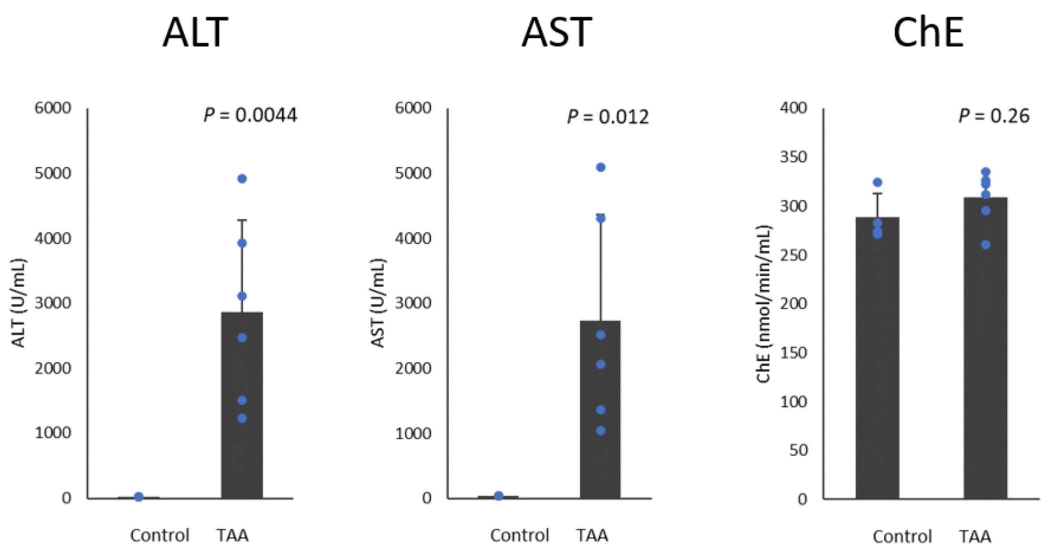

Figure S14. Analysis of blood enzyme activities of blood samples of control mice or TAA-treated mice. Error bars represents S. D. (n = 4 for control mice and n = 6 for TAA-treated mice). *P* value was calculated using Student's t-test. ChE activities were studied using dpNOB (10 μM) incubated with ATC (100 μM) and 1/1000 diluted plasma samples.

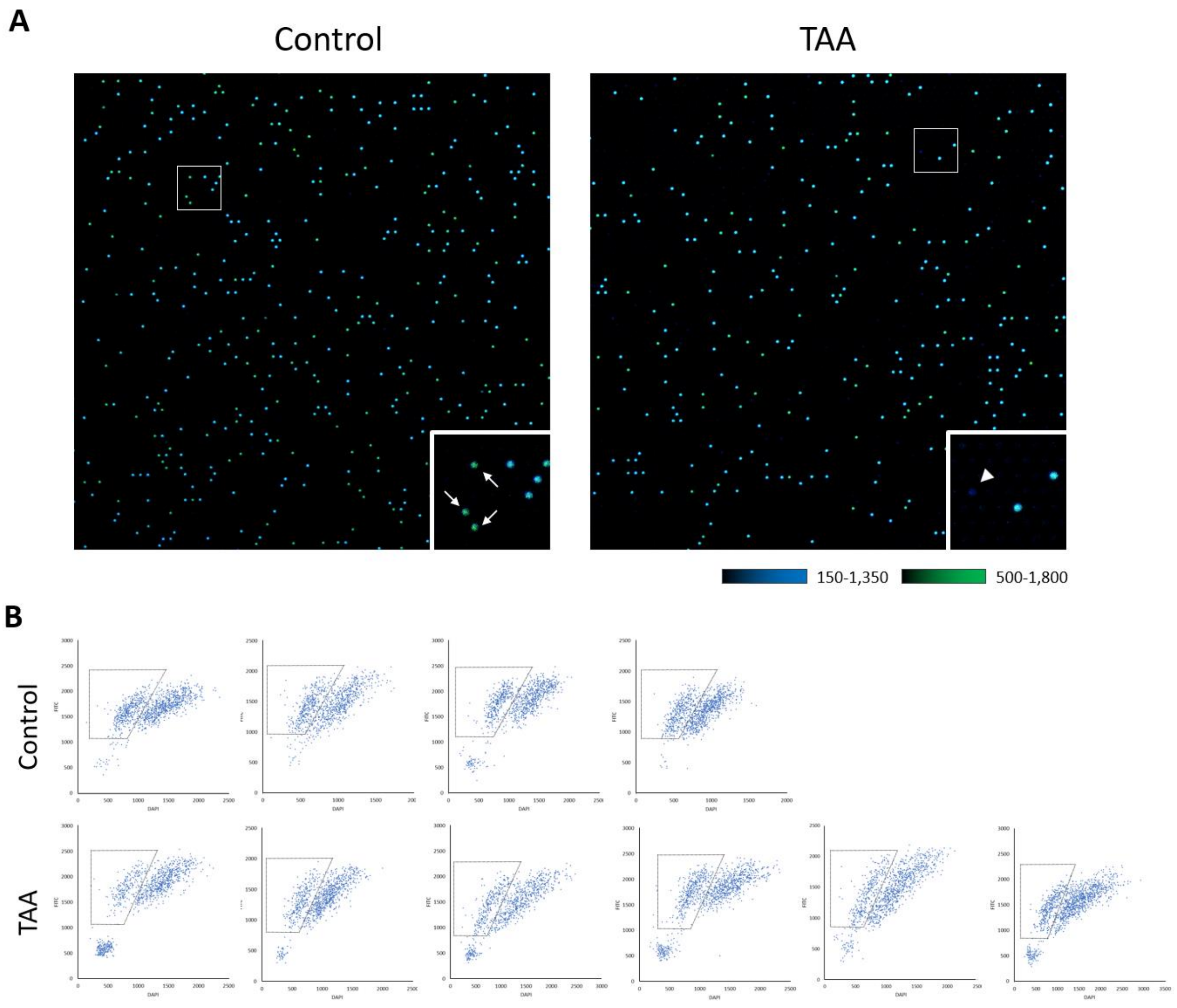

Figure S15. Analysis of blood ChE in control or TAA-treated mice at single-molecule level. (A) Overlayed confocal fluorescence images of microdevice loaded with1/3000 diluted mice plasma samples (control or TAA-treated) mixed with ATC (1 mM), dpNOB (30 μM), and HCCA-Ac (100 μM) and incubated for 2 h. White arrows indicate the enzyme that reacted mainly with ATC + dpNOB (cluster II, BChE), and white arrowheads indicate the enzyme that reacted weakly with HCCA-Ac (cluster III). (B) Scattered plots generated from the analysis of (A).

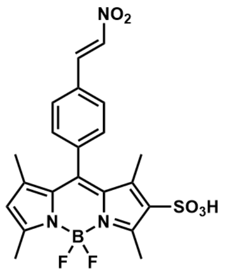

*Preparation of 2-sulfo nitroolefin BODIPY (**1**):* A solution of 8-(*p*-nitroolefin)-BODIPY[15] (7 mg, 0.018 mmol) in acetonitrile (3 mL) was cooled to 0°C, and chlorosulfuric acid (3.13 mg, 0.027 mmol) was added. The mixture was stirred at 0°C for 10 min. The reaction was subsequently quenched with H_2O containing 2 M triethylammonium acetate. The reaction mixture was purified by HPLC (eluent A: H_2O containing 0.1% trifluoroacetic acid; TFA, B: 80 % acetonitrile and 20 % H_2O containing 0.1 % TFA; gradient: A/B = 95/5 to 0/100, 15 min) to obtain **1** (red solid, 1.4 mg, 17% yield). ^{1}H-NMR (400 MHz, DMSO-d_6): δ 8.33 (d, J = 13.7 Hz, 1H), 8.23 (d, J = 13.7 Hz, 1H), 8.06 (d, J = 8.0 Hz, 2H), 7.52 (d, J = 8.0 Hz, 2H), 6.21 (s, 1H), 2.65 (s, 3H), 2.46 (s, 3H), 1.57 (s, 3H), 1.32 (d, 3H). HRMS (ESI$^-$): Calcd. for [M-H]$^-$, 474.11065 ; Found, 474.10862 (-2.04 mDa)

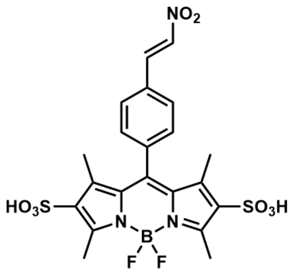

*Preparation of 2,6-sulfo nitroolefin BODIPY (**2**)*: A solution of 8-(*p*-nitroolefin)-BODIPY (10.7 mg, 0.027 mmol) in MeCN (3 mL) was cooled to 0°C, and chlorosulfuric acid (11.04 mg, 6.3 µL, 0.094 mmol) was added. The mixture was stirred at 0°C for 10 min. It was subsequently quenched with H_2O containing 2 M triethylammonium acetate. The reaction mixture was purified by HPLC (eluent A: H_2O containing 0.1% TFA, B: 80 % acetonitrile and 20% H_2O containing 0.1% TFA; gradient: A/B = 95/5 to 0/100, 15 min) to obtain **2** (red solid, 1.8 mg, 12% yield). ^{1}H-NMR (400 MHz, DMSO-d_6): δ 8.34 (d, J = 13.7 Hz, 1H), 8.23 (d, J = 13.7 Hz, 1H), 8.06 (d, J = 7.8 Hz, 2H), 7.52 (d, J = 8.2 Hz, 2H), 2.65 (s, 6H), 1.53 (s, 6H). HRMS (ESI$^-$): Calcd. for [M-H]$^-$, 554.06747; Found, 554.06589 (-1.58 mDa)

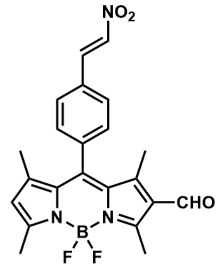

*Preparation of 2-formyl nitroolefin BODIPY (**3**)*: Phosphorous oxychloride (2 mL) was added dropwise to dimethylformamide (2 mL) in an ice batch for 5 min under an Ar atmosphere. The solution was allowed to warm to room temperature and stirred for 30 min. 8-(*p*-nitroolefin)-BODIPY (20 mg, 0.050 mmol) in dichloroethane (10 mL) was added, the temperature raised to 50°C and the reaction mixture was stirred for 30 min. Following this the reaction mixture was allowed to cool to room temperature, quenched with sat. $NaHCO_3$ *aq*. and then aqueous layer was extracted with CH_2Cl_2. The combined organic extracts were washed with brine, dried over anhydrous Na_2SO_4, filtered, and the solvent was removed under reduced pressure. The resulting residue was purified by MPLC (silica gel, 80/20 to 0/100 Hexane/AcOEt) to obtain **3**. (red solid, 12 mg, 56% yield). ^{1}H-NMR (400 MHz, $CDCl_3$): δ 10.02 (s, 1H), 8.08 (d, *J* = 13.7 Hz, 1H), 7.75 (d, *J* = 7.8 Hz, 2H), 7.69 (d, *J* = 13.7 Hz, 1H), 7.44 (d, *J* = 8.2 Hz, 2H), 6.19 (s, 1H), 2.83 (s, 3H), 2.63 (s, 3H), 1.68 (s, 3H), 1.45 (s, 3H). HRMS (ESI$^-$): Calcd. for $[M-H]^-$, 422.14875; Found, 422.15008 (1.33mDa).

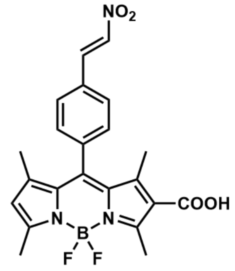

*Preparation of 2-CO_2H nitroolefin BODIPY (**4**)*: **3** (14 mg, 0.033 mmol) was added to a mixture of THF (6 mL) and water (2 mL) followed by the addition of $NaClO_2$ (6 mg, 0.066 mmol) and NH_2SO_3H (29 mg, 0.3 mmol). The reaction mixture was allowed to stir for 30 min, following which it was diluted with AcOEt and washed with sat $Na_2S_2O_3$ *aq*. The organic layer was washed with brine, dried over anhydrous Na_2SO_4, filtered, and the solvent was removed under reduced pressure. The resulting residue was purified by MPLC (silica gel, 80/20 to 0/100 Hexane/AcOEt) to obtain **4**. (red solid, 5 mg, 34% yield). ^{1}H-NMR (400 MHz, DMSO-*d*$_6$): δ 8.33 (d, *J* = 13.7 Hz, 1H), 8.21 (d, *J* = 13.7 Hz, 1H), 8.04 (d, *J* = 8.2 Hz, 2H), 7.53 (s, 2H), 6.35 (s, 1H), 2.64 (s, 3H), 2.48 (s, 3H), 1.57 (s, 3H), 1.34 (s, 3H). HRMS (ESI$^-$): Calcd. for $[M-H]^-$, 438.14367; Found, 438.14181 (-1.86 mDa)

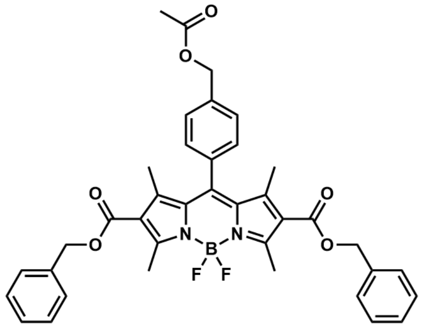

*Preparation of 2,6-diCO$_2$Bzl acetoxymethyl BODIPY (**5**)*: 2,4-Dimethyl-1*H*-pyrrole-3-carboxylic acid ethyl ester (250 mg, 1.09 mmol) and (4-formylphenyl)methyl acetate (96 mg, 0.54 mmol) were dissolved in CH_2Cl_2 (100 mL). TFA (0.1 mL) was added under an Ar atmosphere, and the solution was stirred at room temperature for 12 h. 2,3-Dichloro-5,6-dicyano-*p*-benzoquinone (DDQ; 122 mg, 0.54 mmol) was added, and after 30 min of stirring, the reaction was washed with brine three times, dried over Na_2SO_4, filtered, and evaporated. The remaining red solid was purified on a filtration column (alumina). Acquired crude dipyrromethene was dissolved in toluene and *N*,*N*-diisopropyl-*N*-ethylamine (DIEA; 2 mL). BF_3-OEt_2 (1.5 mL) was added dropwise under an Ar atmosphere, and the solution was stirred at room temperature for 1 h. Then AcOEt was added, and the mixture was washed with brine, dried over Na_2SO_4, filtered, and evaporated. The resulting residue t was purified by MPLC (silica gel, 80/20 to 0/100 Hexane/AcOEt) to obtain **5** (orange solid, 120 mg, 33% yield). ^{1}H-NMR (400 MHz, $CDCl_3$): δ 7.51-7.24 (m, 14H), 5.26 (s, 4H), 5.21 (s, 2H), 2.82 (s, 6H), 2.17 (s, 3H), 1.64 (s, 6H). ^{13}C-NMR (101 MHz, $CDCl_3$): δ 170.9, 164.1, 160.0, 147.9, 145.5, 138.0, 135.9, 134.1, 131.5, 129.0, 128.7, 128.4, 128.0, 122.3, 66.3, 65.5, 21.1, 15.3, 14.0. HRMS (ESI$^+$): Calcd. for $[M+H]^+$, 665.26345 ; Found, 665.26588 (+2.43 mDa)

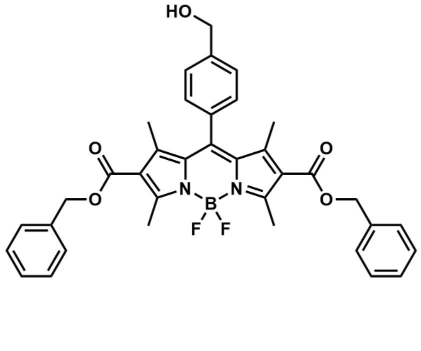

*Preparation of 2,6-diCO$_2$Bzl hydroxymethyl BODIPY (**6**)*: **5** (200 mg, 0.298 mmol) was dissolved in in CH_2Cl_2/methanol, 2:1 (20 mL). 2N NaOH *aq*. (2 mL) was added, and the

solution was stirred at room temperature for 3 h. The reaction mixture was diluted with CH_2Cl_2, washed with 2N HCl *aq*. and brine, dried over anhydrous Na_2SO_4, filtered, and the solvent was removed under reduced pressure. The resulting residue was purified by MPLC (silica gel, 80/20 to 0/100 Hexane/AcOEt) to obtain **6** (orange solid, 170 mg, 93% yield). ^{1}H-NMR (400 MHz, $CDCl_3$): δ 7.54-7.24 (m, 14H), 5.31 (s, 1H), 5.27 (s, 4H), 4.84 (s, 2H), 2.83 (s, 6H), 1.65 (s, 6H). ^{13}C-NMR (101 MHz, $CDCl_3$): δ 164.1, 159.9, 148.0, 145.9, 142.8, 135.9, 133.4, 131.6, 128.7, 128.4, 127.9, 127.8, 122.2, 66.3, 64.7, 15.3, 14.1. HRMS (ESI$^+$): Calcd. for [M+H]+ , 623.25288; Found, 623.25423 (1.35 mDa)

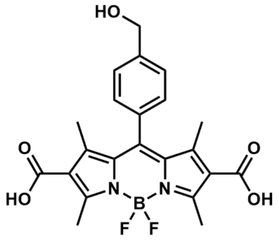

*Preparation of 2,6-diCO$_2$H hydroxymethyl BODIPY (**7**)*: **6** (40 mg, 0.64 mmol) was dissolved in CH_2Cl_2/methanol, 2:1 (20 mL). After addition of a small amount of 10% Pd-C, the mixture was stirred under a H_2 atmosphere for 3 h. When TLC monitoring (silica; CH_2Cl_2-0.1% (v/v) AcOH) showed that the formation of the product was complete, the Pd-C was filtered off, washed with brine, dried over anhydrous Na_2SO_4, filtered, and the solvent was removed under reduced pressure. The resulting residue was purified by HPLC (eluent A: H_2O containing 100 mM triethylammonium acetate, B: 80 % acetonitrile and 20 % H_2O containing 100 mM triethylammonium acetate; gradient: A/B = 95/5 to 0/100, 15 min). Obtained solution was desalted, evaporated under reduced pressure and freeze dried to obtain **7**. (red solid, 8 mg, 28% yield). ^{1}H-NMR (400 MHz, DMSO-d_6): δ 7.53 (d, J = 8.2 Hz, 2H), 7.36 (d, J = 8.2 Hz, 2H), 5.42 (s, 1H), 4.63 (s, 2H), 2.70 (s, 6H), 1.59 (s, 6H). HRMS (ESI$^-$): Calcd. for [M-H]$^-$, 441.14333; Found, 441.14091 (-2.42 mDa)

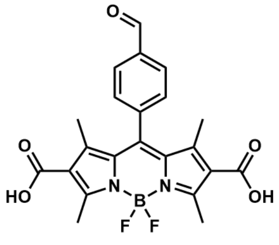

*Preparation of 2,6-diCO$_2$H formyl BODIPY (**8**)*: To a solution of **7** (8 mg, 0.018 mmol) in MeCN (2 mL) containing 10% DMSO, Dess-Martin periodinane (6.1 mg, 0.027 mmol) was

added. The mixture was stirred at room temperature for 3 h, and evaporated. A crude **8** was used in the next step without further purification.

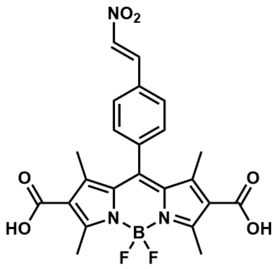

*Preparation of 2,6-diCO$_2$H nitroolefin BODIPY (**9**):* A mixture of nitromethane (10 µL, 0.2 mmol) and **8** in methanol (0.5 mL) was stirred at 0˚C. 2N NaOH *aq.* (1 mL) was added over a period of 30 min. The stirring was continued for another 30 min at 0˚C. The mixture was diluted with water (3 mL) and poured over crushed ice containing 3 mL conc. HCl. After 30 min of stirring, the reaction was evaporated. The remaining liquid was purified by HPLC (eluent A: H_2O containing 100 mM triethylammonium acetate, B: 80 % acetonitrile and 20 % H_2O containing 100 mM triethylammonium acetate; gradient: A/B = 95/5 to 0/100, 15 min). Obtained solution was desalted, evaporated under reduced pressure and freeze dried to obtain **9** (red solid, 3.4 mg, 3.3% yield in 2 steps). ^{1}H-NMR (400 MHz, CD_3OD): δ 8.15 (d, *J* = 13.7 Hz, 1H), 8.04 (d, *J* = 13.7 Hz, 1H), 7.93 (d, *J* = 7.8 Hz, 2H), 7.50 (d, *J* = 7.8 Hz, 2H), 2.75 (s, 6H), 1.68 (s, 6H). HRMS (ESI$^-$): Calcd. for $[M-H]^-$, 482.13350; Found, 482.13229 (-1.21 mDa)

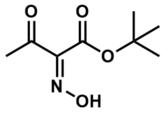

P*reparation of tert-butyl 2-hydroximino-3-oxobutyrate (**10**)*: *tert*-Butyl 3-oxobutanoate (8 mL, 48 mmol) was dissolved in acetic acid (20 mL), and the mixture was cooled in an ice bath. Sodium nitrite (3.6 g, 52 mmol) was added over 30 min while the temperature was kept under 15°C. The cold bath was removed, and the mixture was allowed to stir for 3.5 h at room temperature. The reaction mixture was diluted with CH_2Cl_2, washed with sat. $NaHCO_3$ *aq.* and brine, dried over anhydrous Na_2SO_4, filtered, and the solvent was removed under reduced pressure. A crude **10** was used in the next step without further purification.

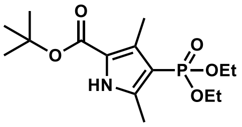

*Preparation of Compound **11***: To a stirred mixture of diethyl (2-oxopropyl)phosphonate (3.5 mL, 20 mmol), zinc dust (1.3 g, 20 mmol) was added in portion. The mixture was heated to 60°C. At this temperature, the crude **10** (3.6 g, 20 mmol) in acetic acid (3 mL) precipitated slowly. The temperature was then increased to 75°C and kept for 1 h. The reaction mixture was diluted with CH_2Cl_2, washed with brine, dried over anhydrous Na_2SO_4, filtered, and the solvent was removed under reduced pressure. The resulting residue was purified by HPLC (eluent A: H_2O containing 0.1% TFA, B: 80 % acetonitrile and 20 % H_2O containing 0.1 % TFA; gradient: A/B = 95/5 to 0/100, 15 min) to obtain **11** (pale brown oil, 458 mg, 7% yield). ^{1}H-NMR (400 MHz, $CDCl_3$): δ 9.01 (s, 1H), 4.12-4.01 (m, 4H), 2.49 (s, 2H), 2.41 (s, 2H), 1.57 (s, 9H), 1.33-1.30 (m, 6H). ^{13}C-NMR (101 MHz, $CDCl_3$): δ 160.9, 140.5, 131.3, 120.6, 107.5, 81.4, 61.7, 28.5, 16.3, 13.5, 11.8. HRMS (ESI$^-$): Calcd. for $[M-H]^-$, 330.14703; Found, 330.14519 (-1.85 mDa)

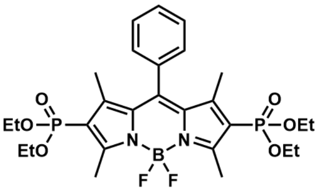

Preparation of 2,6-diPO$_3$Et$_2$-BODIPY (12): **11** (214 mg, 0.64 mmol) and benzaldehyde (35 µL, 0.32 mmol) were dissolved in CH_2Cl_2 (100 mL). Trifluoroacetic acid (0.1 mL) was added under an Ar atmosphere, and the solution was stirred at room temperature for 12 h. DDQ (72.6 mg, 0.32 mmol) was added, and after 30 min of stirring, the reaction was washed with brine three times, dried over Na_2SO_4, filtered, and evaporated. The remaining red solid was purified on a filtration column (alumina). Acquired crude dipyrromethene was dissolved in toluene and DIEA (2 mL). BF_3-OEt_2 (1.5 mL) was added dropwise under an Ar atmosphere, and the solution was stirred at room temperature for 1 h. Then AcOEt was added, and the mixture was washed with brine, dried over Na_2SO_4, filtered, and evaporated. The crude product was purified by MPLC (silica gel, 80/20 to 0/100 Hexane/AcOEt) to afford **12** (orange solid, 32 mg, 16% yield). ^{1}H-NMR (400 MHz, $CDCl_3$): δ 7.54 (m, 3H), 7.26 (m, 2H), 4.07 (m, 8H), 2.81 (s, 6H), 1.63 (s, 6H), 1.30 (m, 12H). ^{13}C-NMR (101 MHz, $CDCl_3$): d 161.2, 161.0, 151.3, 151.2, 145.4, 134.2, 132.4, 132.2, 129.9, 129.8, 127.5, 118.9, 116.9, 61.8,

61.7, 16.4, 14.7, 13.9. HRMS (ESI$^+$): Calcd. for $[M+H]^+$, 597.22662; Found, 597.22734 (0.72 mDa)

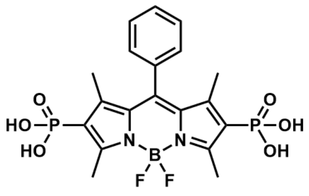

*Preparation of 2,6-diPO$_3$H$_2$-BODIPY (**13**)*: **12** (32 mg, 0.05 mmol) was dissolved in CH_2Cl_2 (2 mL). Trimethylsilyl iodide (66 µL, 0.45 mmol)) was added under an Ar atmosphere, and the solution was stirred at room temperature for 15 min. Sat. $Na_2S_2O_3$ *aq*. was added. After 30 min of stirring, the reaction was evaporated. The remaining liquid was purified by HPLC (eluent A: H_2O containing 100 mM triethylammonium acetate, B: 80 % acetonitrile and 20 % H_2O containing 100 mM triethylammonium acetate; gradient: A/B = 95/5 to 0/100, 15 min). Obtained solution was desalted, evaporated under reduced pressure and freeze dried to obtain **13** (orange solid, 4 mg, 16% yield). ^{1}H-NMR (400 MHz, DMSO-d_6): δ 7.51- 7.22 (m, 4H), 4.62 (s, 2H), 2.43 (s, 6H), 1.48 (s, 6H)

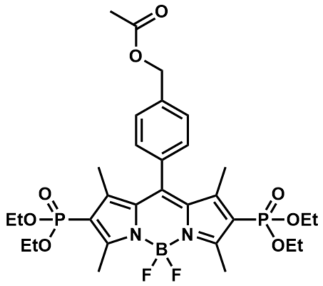

*Preparation of 2,6-diPO$_3$Et$_2$ acetoxymethyl BODIPY (**14**)*: **11** (379 mg, 1.15 mmol) and (4-formylphenyl)methyl acetate (101.5 mg, 0.57 mmol) were dissolved in CH_2Cl_2 (200 mL). TFA (0.1 mL) was added under an Ar atmosphere, and the solution was stirred at room temperature for 12 h. DDQ (129.4 mg, 0.57 mmol) was added, and after 30 min of stirring, the reaction was washed with brine three times, dried over Na_2SO_4, filtered, and evaporated. The remaining red solid was purified on a filtration column (alumina). Acquired crude dipyrromethene was dissolved in toluene and DIEA (2 mL). BF_3-OEt_2 (1.5 mL) was added dropwise under an Ar atmosphere, and the solution was stirred at room temperature for 1 h. Then AcOEt was added, and the mixture was washed with brine, dried over Na_2SO_4, filtered, and evaporated. The crude product was purified by MPLC (silica gel, 80/20 to 0/100 Hexane/AcOEt) to obtain **14** (orange solid, 158 mg, 18% yield). ^{1}H-NMR (400 MHz, $CDCl_3$):

δ 7.53 (d, J = 8.2 Hz, 2H), 7.28 (d, J = 8.2 Hz, 2H), 5.20 (s, 2H), 4.16-3.98 (m, 8H), 2.82 (s, 6H), 2.16 (s, 3H), 1.63 (s, 6H), 1.32-1.28(m, 12H). ^{13}C-NMR (101 MHz, $CDCl_3$): δ 161.2, 161.0, 151.3, 151.2, 145.5, 143.4, 133.0, 132.5, 132.3, 127.9, 127.6, 118.7, 116.7, 64.3, 61.9, 61.8, 29.8, 16.4, 16.3, 14.7, 14.0. HRMS (ESI$^+$): Calcd. for $[M+H]^+$, 669.24775; Found, 669.24394 (-3.80 mDa)

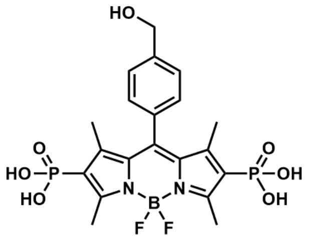

*Preparation of 2,6-diPO$_3$H$_2$ hydroxymethyl BODIPY (**15**)*: **14** (50 mg, 0.074 mmol) was dissolved in CH_2Cl_2 (2 mL). Trimethylsilyl iodide (97 µL, 0.66 mmol) was added under an Ar atmosphere, and the solution was stirred at room temperature for 15 min. The mixture was quenched with sat. $Na_2S_2O_3$ *aq*, and then 2N NaOH *aq*. (1 mL) was added. After 30 min of stirring, the reaction was evaporated. The remaining liquid was purified by HPLC (eluent A: H_2O containing 100 mM triethylammonium acetate, B: 80 % acetonitrile and 20 % H_2O containing 100 mM triethylammonium acetate; gradient: A/B = 95/5 to 0/100, 15 min). Obtained solution was desalted, evaporated under reduced pressure and freeze dried to obtain **15** (orange solid, 5 mg, 14% yield). ^{1}H-NMR (400 MHz, DMSO-d_6): δ 7.51 (d, J = 8.2 Hz, 2H), 7.22 (m, 2H), 4.62 (s, 2H), 2.43 (s, 6H), 1.48 (s, 6H). HRMS (ESI$^-$): Calcd. for $[M-H]^-$, 513.09634; Found, 513.09675 (0.41 mDa)

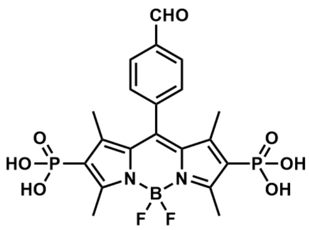

*Preparation of 2,6-diPO$_3$H$_2$ formyl BODIPY (**16**)*: To a solution of **15** (5 mg, 0.01 mmol) in MeCN (2 mL) containing 10% DMSO, Dess-Martin periodinane (6.2 mg, 0.015 mmol) was added. The mixture was stirred at room temperature for 3 h, and evaporated to obtain a crude **16**, which was used in the next step without further purification.

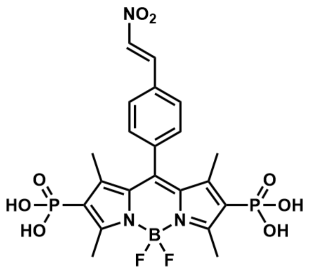

*Preparation of 2,6-diPO$_3$H$_2$ nitroolefin BODIPY (**17**)*: A mixture of nitromethane (1 µL, 0.02 mmol) and **16** in methanol (0.5 mL) was stirred at 0°C. 2N NaOH *aq*. (1 mL) was added over a period of 30 min. The stirring was continued for another 30 min at 0°C. The mixture was diluted with water (3 mL) and poured over crushed ice containing 3 mL conc. HCl. After 30 min of stirring, the reaction was evaporated. The remaining liquid was purified by HPLC (eluent A: H_2O containing 100 mM triethylammonium acetate, B: 80 % acetonitrile and 20 % H_2O containing 100 mM triethylammonium acetate; gradient: A/B = 95/5 to 0/100, 15 min). Obtained solution was desalted, evaporated under reduced pressure and freeze dried to obtain **17** (orange solid, 1.2 mg, 20% yield in 2 steps). ^{1}H-NMR (400 MHz, CD_3OD): δ 8.16 (d, J = 13.7 Hz, 1H), 8.04 (d, J = 13.7 Hz, 1H), 7.93 (d, J = 8.2 Hz, 2H), 7.49 (d, J = 7.8 Hz, 2H), 2.72 (s, 6H), 1.67 (s, 6H). HRMS (ESI$^-$): Calcd. for [M-H]$^-$, 554.08650; Found, 554.08845 (1.95 mDa)
